# Supplementary material for: Adaptive foraging of pollinators fosters gradual tipping under resource competition and rapid environmental change
Source: PLoS Comput Biol. 2024 Jan 9;20(1):e1011762. doi: 10.1371/journal.pcbi.1011762 (PMC10802948; doi:10.1371/journal.pcbi.1011762)
Supplement: S1 Text — The text with supporting information contains additional computational experiments and algorithms. It is divided into five sections. Section A. Simulation set-up. Includes: Table A. Default model parameters. The default parameter values and ranges used in all simulations, unless otherwise specified. AF = Adaptive Foraging, ∼ U(⋅) = drawn from a uniform distribution at the beginning of each simulation. Section B. Network generation. Describes the network generation algorithm and contains: Fig A. Adjacency matrix of a nested network and forbidden links Adjacency matrix of a pollinator network on the left with the corresponding forbidden links matrix on the right. Black squares denote the presence of a link. The connectance is 0.15 and the fraction of forbidden links is 0.3. There is a clear difference visible between generalist species and specialist species, in the sense that there are a few species with high connectivity and many with low connectivity. Section C. Dependence of hysteresis on resource congestion and adaptation. Contains various additional computation experiments. Fig B. Hysteresis for increasing resource congestion q for the non-adaptive model. Equilibrium abundance of pollinator species as a function of the drivers of decline dA for increasing resource congestion q for the non-adaptive model. The blue lines show the equilibrium trajectory for increasing dA and the orange lines show the equilibrium trajectory for decreasing dA. See Table A for the parameters used. Fig C. Hysteresis for increasing resource congestion q for the adaptive model with ν = 0.8. Equilibrium abundance of pollinator species as a function of the drivers of decline dA for increasing resource congestion q for the adaptive model with ν = 0.8. The blue lines show the equilibrium trajectory for increasing dA and the orange lines show the equilibrium trajectory for decreasing dA. See Table A for the parameters used. Fig D. Hysteresis for increasing resource congestion q for the adapti [file pcbi.1011762.s001.pdf]

SUPPORTING INFORMATION

Adaptive Foraging of Pollinators Fosters Gradual Tipping under Resource Competition and Rapid Environmental Change — Supporting Information

Sjoerd Terpstra[1,2,3] | Flávia M. D. Marquitti[4,5] | Vítor V. Vasconcelos[6,3*]

*Correspondence: v.v.vasconcelos@uva.nl. 1 - Graduate School of Informatics, University of Amsterdam, The Netherlands. 2 - Institute for Marine and Atmospheric Research Utrecht, Utrecht University, Utrecht, The Netherlands. 3 - Institute for Advanced Study, University of Amsterdam, The Netherlands. 4 - Instituto de Física 'Gleb Wataghin' & Programa de Pós Graduação em Ecologia, Universidade Estadual de Campinas, Brazil. 5 - International Centre for Theoretical Physics - South American Institute for Fundamental Research (ICTP-SAIFR), Brazil. 6 - Informatics Institute, University of Amsterdam, The Netherlands.

1 # A | SIMULATION SET-UP

TABLE A The default parameter values and ranges used in all simulations, unless otherwise specified. AF = Adaptive Foraging, $\sim U(\cdot)$ = drawn from a uniform distribution at the beginning of each simulation.

Parameter	Default Value	Explanation
S^P	15	number of plant species
S^A	35	number of pollinator species
FL	0.3	forbidden links (fraction)
D	0.15	connectance (fraction)
N	0.6	nestedness
r_i	$r_i \sim U(0.1, 0.35)$	intrinsic growth rate
h_i	$h_i \sim U(0.15, 0.3)$	mutualism saturation
C_{ii}	$C_{ii} \sim U(0.8, 1.1)$	intraspecific competition
C_{ij}	$C_{ij} \sim U(0.01, 0.05)$	interspecific competition
β^P	$\beta^P_{ij} \sim U(0.8, 1.2)$	plant matching matrix
β^A	$\beta^A_{ij} \sim U(0.8, 1.2)$	pollinator matching matrix
α	$\sum_i \alpha_{ij} = 1$	foraging effort matrix
η	0.5	β normalisation exponent
μ	0.0001	migration rate
q	0	resource congestion
ν	1	AF strength
d_A	0	driver of decline
d_A^{init}	0	initial driver of decline
λ	0	rate of change of d_A

B | NETWORK GENERATION

The mutualistic interaction network is a bipartite network, described by a binary adjacency matrix M.[1] The important parameters for the network are nestedness (N), connectance (D), fraction of forbidden links (FL), number of plant species (S^P), and number of pollinator species (S^A). The nestedness is a measure of the shared interaction between species. It is calculated by taking the sum of shared interactions between all pairs of species, and normalising it so that $0 \leq N \leq 1$

$$N = \frac{N_p + N_a}{\frac{S^P(S^P-1)}{2} + \frac{S^A(S^A-1)}{2}}\,, \tag{1}$$

$$N_p = \sum_{i<j}^{S^P} \frac{n_{ij}}{\min(n_i, n_j)}, \quad N_a = \sum_{i<j}^{S^A} \frac{n_{ij}}{\min(n_i, n_j)}\,, \tag{2}$$

with n denoting the number of interactions (Bastolla et al., 2009). D is the fraction of links that exists in the network of all possible links. For a bipartite network, the total number of possible links is equal to $S^P \cdot S^A$. Thus, the connectance D is defined as

$$D = \frac{\sum_{ij} M_{ij}}{S^P \cdot S^A}\,. \tag{3}$$

The fraction of forbidden links FL is the fraction of all links that cannot exist due to a phenological mismatch between species (Olesen et al., 2011).

The networks representing the mutualistic interaction between plant and pollinator species were created in three steps, based on the algorithms described by Lever et al. (2014) and Bastolla et al. (2009). First, the forbidden links were uniformly sampled from all possible links. Then, the connected links were obtained by uniformly sampling all possible links, minus those that were forbidden links. This gave a randomly connected bipartite network represented by an adjacency matrix. If not all species had at least one interaction, the forbidden and connected links were redrawn.

Second, the random network was transformed into a nested network through an iterative procedure which was repeated until the targeted nestedness was reached. If after 10^5 iterations the required nestedness was not reached, the network was discarded and a new one was generated. During each iteration, a random plant and a random pollinator species were selected. Then, either another plant or another pollinator species was randomly selected. If this time a plant species was selected, the interaction between the first plant and pollinator species was rewired to an interaction between the pollinator and the new plant species, given that the following criteria were met: the first plant species had at least one connection left after rewiring, the new connection was not a forbidden link, and the newly selected plant species had more interactions than the first plant species. The algorithm was the same when the third species selected was a pollinator, except that the roles of all species were swapped.

The third condition—that the species that accepts the new interaction needs to have a higher degree than the original species—ensured that well-connected species (generalists) obtained more interactions, while less connected species lost most of their interactions, thereby generating a power-law distribution of node degrees. Each species always kept at least one interaction, so no loose species (nodes) existed in the network.

Finally, the resulting nested network was checked to see if it had a single component (that is, there were no disconnected groups of species). A breadth-first search (BFS) algorithm—adapted from Networkx to work for bipartite

[1] M is equal to the binary conversion of the matching matrix β. To be more exact: the values in the β matrix are sampled based on this binary matrix M.

networks—was used for this (Hagberg et al., 2008). If there were multiple components in the network, the network was discarded and a new one was generated.

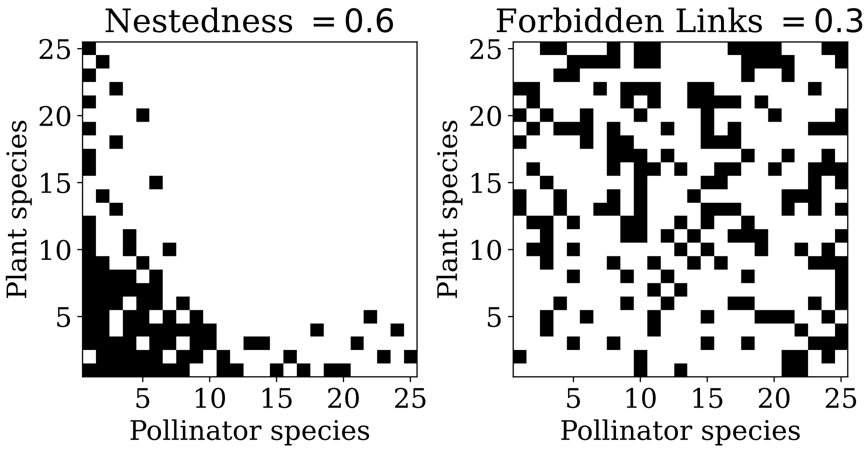

FIGURE A Adjacency matrix of a nested network and forbidden links. Adjacency matrix of a pollinator network on the left with the corresponding forbidden links matrix on the right. Black squares denote the presence of a link. The connectance is 0.15 and the fraction of forbidden links is 0.3. There is a clear difference visible between generalist species and specialist species, in the sense that there are a few species with high connectivity and many with low connectivity.

Figure A shows an example of a network with $D = 0.15$, $FL = 0.3$, and $N = 0.6$. The adjacency matrix shows that there are generalist and specialist species present. The generalists have many connections (many black squares for the species in the matrix) and the specialists have only few connections (one black square for the species in the matrix). The forbidden links showcase the links that are not allowed to be present in the network which are used for generating the nested networks.

C | DEPENDENCE OF HYSTERESIS ON RESOURCE CONGESTION AND ADAPTATION

Figures SB, SC, SD and SE show the hysteresis as function of resource congestion q for $\nu = 1$, $\nu = 0.8$, $\nu = 0.7$ and $\nu = 0.6$ respectively. For all models, the bistability region disappeared for higher q. At the same time, higher adaptation ν increased the spread in abundances and the spread of values of drivers of decline d_A at which each species went extinct.

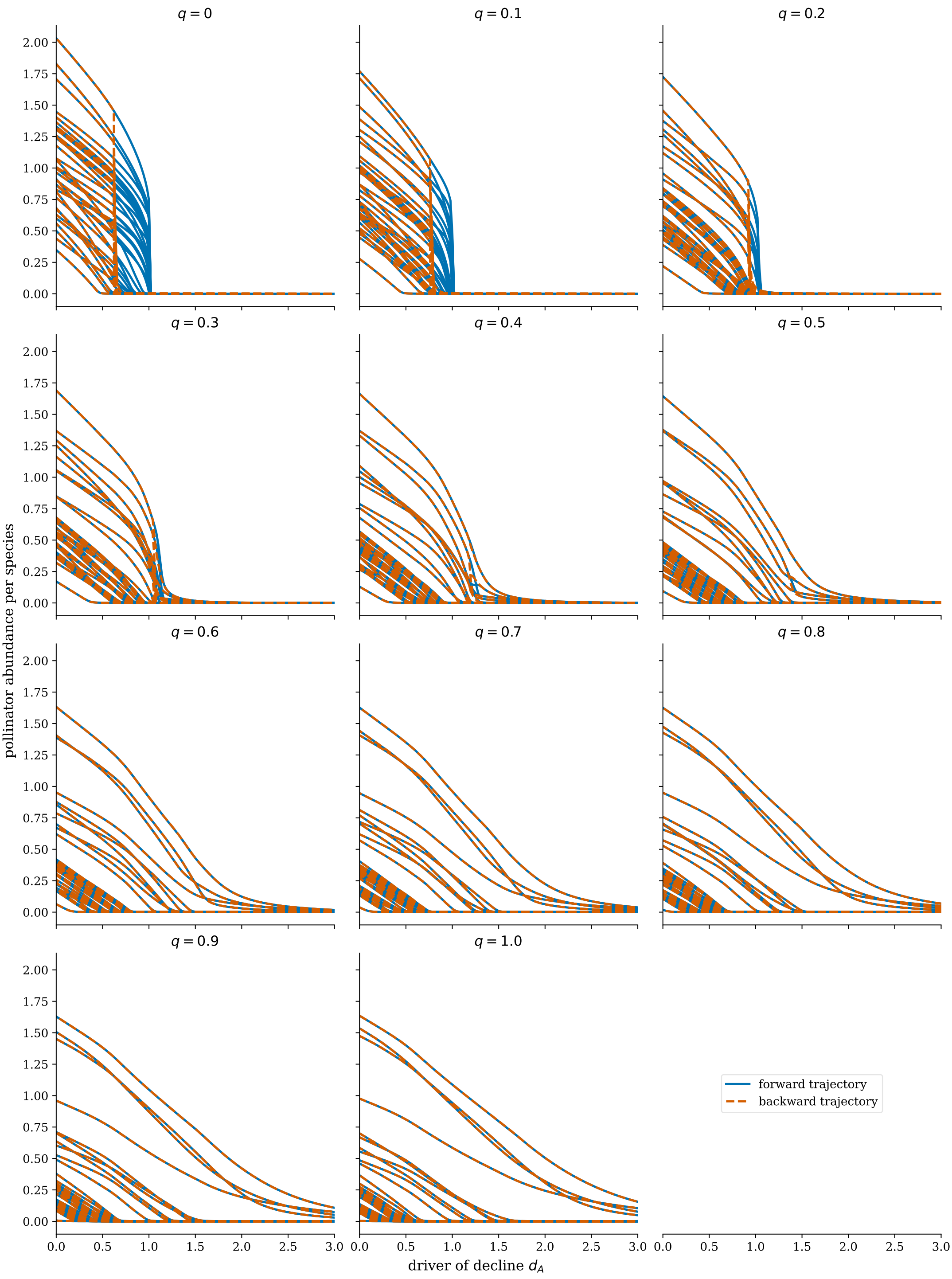

FIGURE B Hysteresis for increasing resource congestion q for the non-adaptive model. Equilibrium abundance of pollinator species as a function of the drivers of decline d_A for increasing resource congestion q for the non-adaptive model. The blue lines show the equilibrium trajectory for increasing d_A and the orange lines show the equilibrium trajectory for decreasing d_A. See Table SA for the parameters used.

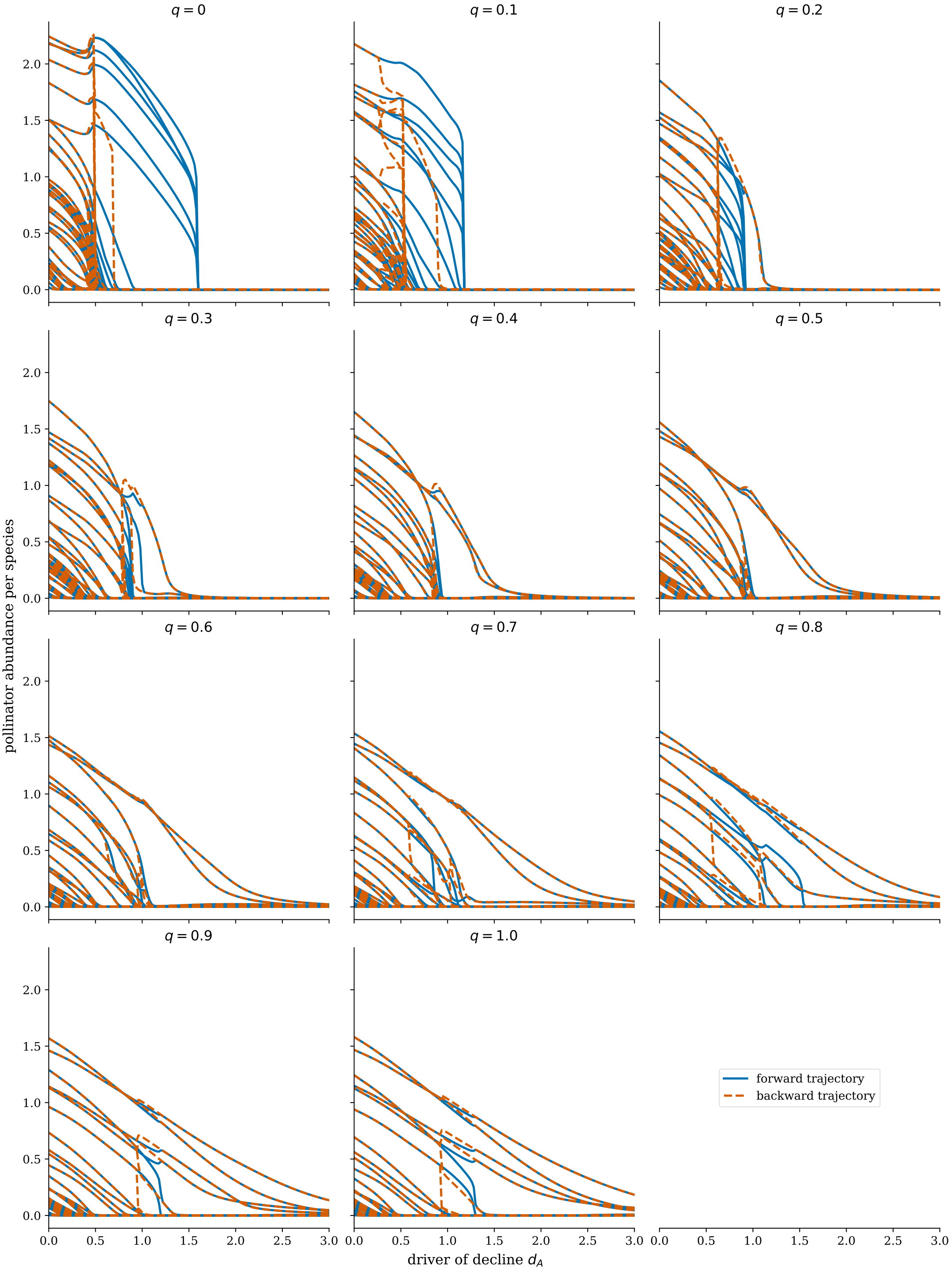

FIGURE C Hysteresis for increasing resource congestion q for the adaptive model with $\nu = 0.8$. Equilibrium abundance of pollinator species as a function of the drivers of decline d_A for increasing resource congestion q for the adaptive model with $\nu = 0.8$. The blue lines show the equilibrium trajectory for increasing d_A and the orange lines show the equilibrium trajectory for decreasing d_A. See Table SA for the parameters used.

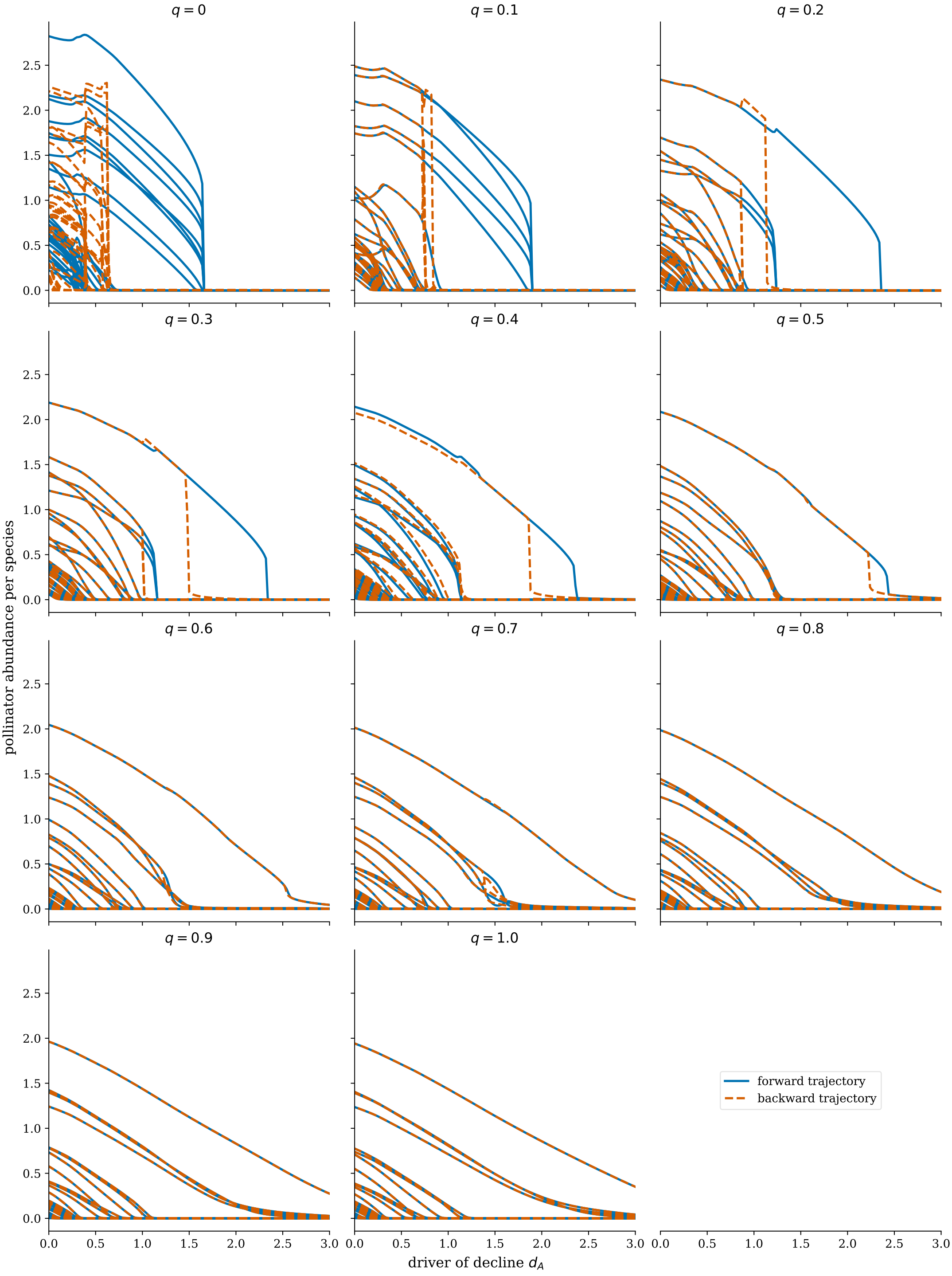

FIGURE D Hysteresis for increasing resource congestion q for the adaptive model with $\nu = 0.7$. Equilibrium abundance of pollinator species as a function of the drivers of decline d_A for increasing resource congestion q for the adaptive model with $\nu = 0.7$. The blue lines show the equilibrium trajectory for increasing d_A and the orange lines show the equilibrium trajectory for decreasing d_A. See Table SA for the parameters used.

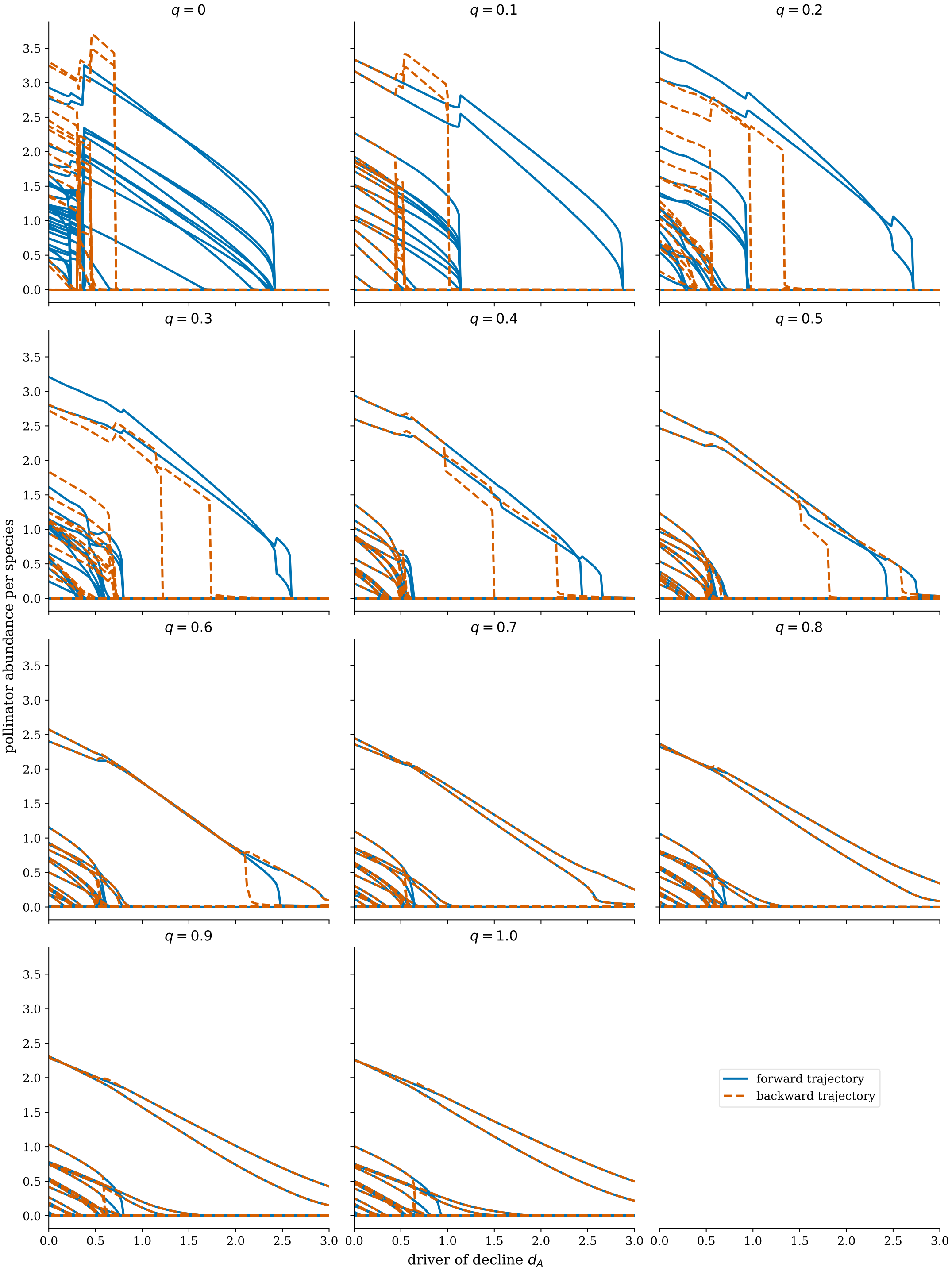

FIGURE E Hysteresis for increasing resource congestion q for the adaptive model with $\nu = 0.6$. Equilibrium abundance of pollinator species as a function of the drivers of decline d_A for increasing resource congestion q for the adaptive model with $\nu = 0.6$. The blue lines show the equilibrium trajectory for increasing d_A and the orange lines show the equilibrium trajectory for decreasing d_A. See Table SA for the parameters used.

D | DISTRIBUTION OF POLLINATOR PERSISTENCE

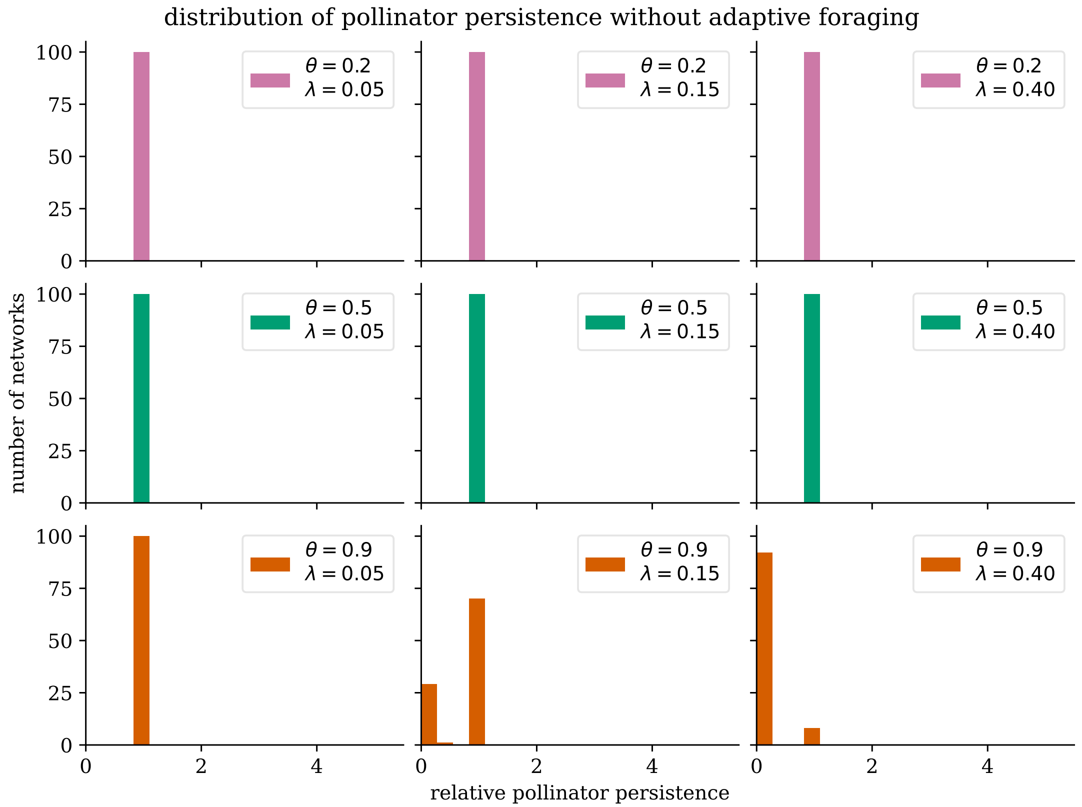

FIGURE F The full distribution of relative pollinator abundance for three different rates of change λ accompanying Figure 2A in the paper (no adaptive foraging). θ is the fraction of the point of collapse d_A at which point the relative pollinator persistence is measured. The distributions are bimodal around 0 and 1 which indicates that there is an abrupt collapse of networks at increasing rates of change. See Table SA for the parameters used.

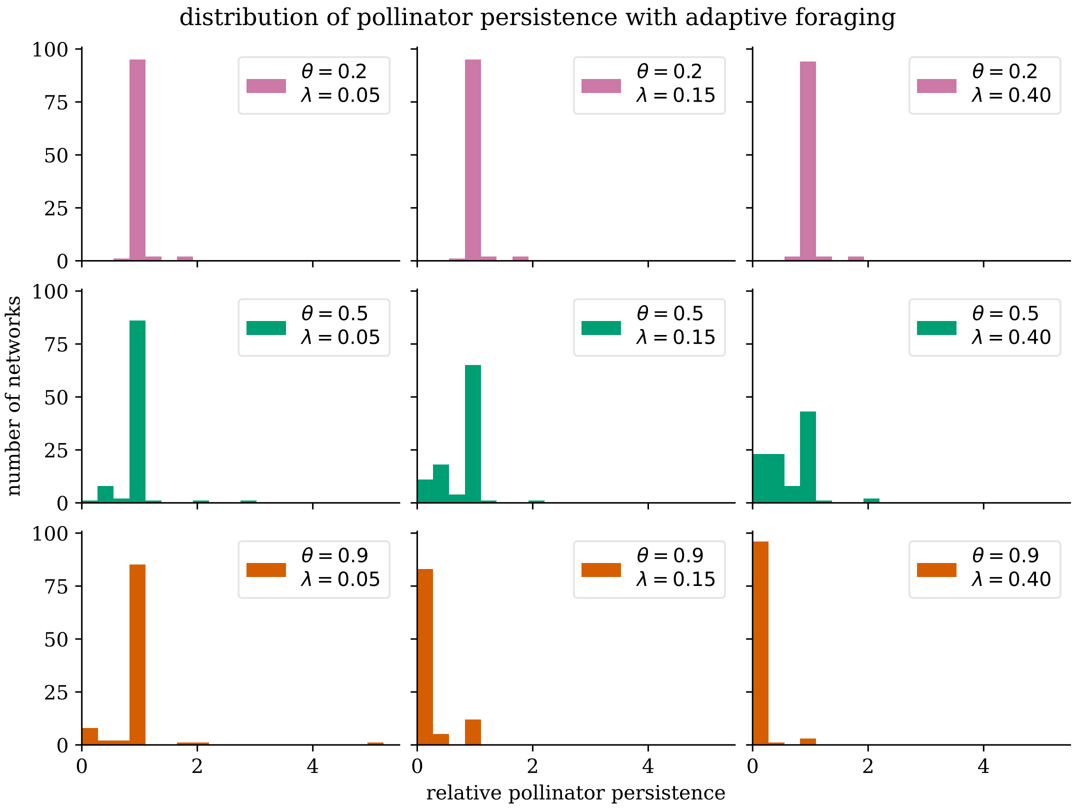

FIGURE G The full distribution of relative pollinator abundance for three different rates of change λ accompanying Figure 2A in the paper (with adaptive foraging). θ is the fraction of the point of collapse d_A at which point the relative pollinator persistence is measured. The distributions are mainly bimodal around 0 and 1. However, some networks have a persistence between 0 and 1, indicating partial collapse due to the rate of change. Furthermore, there are a few networks with pollinator persistence significantly above 1, indicating nonlinear effects where sometimes individual networks can profit from higher rates of change. See Table SA for the parameters used.

E | SENSITIVITY ANALYSIS

To discern the influence of key model parameters on the feasibility of the solution, we performed a Sobol sensitivity analysis (Sobol, 2001; Saltelli, 2002; Saltelli et al., 2010), which is a global, variance-based sensitivity analysis. This sensitivity analysis relates the variance in the outcome of the model to the uncertainty of the inputs by calculating the Sobol indices. The first-order index represents the variance caused by varying a single parameter and the second-order index represents the variance caused by varying two parameters together. The total-order index takes into account all interactions of a parameter with the other parameters, giving the total influence of a parameter on variance in the model output. The sensitivity analysis was performed using the SALib library (Herman and Usher, 2017).

| Sensitivity analysis on the feasibility of networks

Figures SH, SI, SJ, SK and SL show the sensitivity analysis on the feasibility of the networks and abundance of plant and pollinator species. The feasibility is defined as the number of species alive in equilibrium under zero external stressors (i.e., the driver of decline $d_A = 0$). Per definition, each result is only significant if the confidence interval does not include the sensitivity index of zero.

The sensitivity analysis was performed over five parameters, see Table SB. The number of samples per parameter was 512. In total, this led to a total Saltelli sample size of 6144. The simulation was run ten times for each sample, each time with a different network. Since the aim of this sensitivity analysis was to look at the feasibility of the networks, the networks were not tested on feasibility during their generation.

TABLE B Parameters and their value ranges used for the sensitivity analysis on the feasibility of networks, and plant and pollinator abundances. The fixed parameters can be found in Table SA. AF = Adaptive Foraging.

Parameter	Range of values	Explanation
q	$[0, 1]$	resource congestion
N	$[0.2, 0.7]$	nestedness
D	$[0.15, 0.35]$	connectance
μ	$[0.00001, 0.001]$	migration rate
ν	$[0, 1]$	AF strength

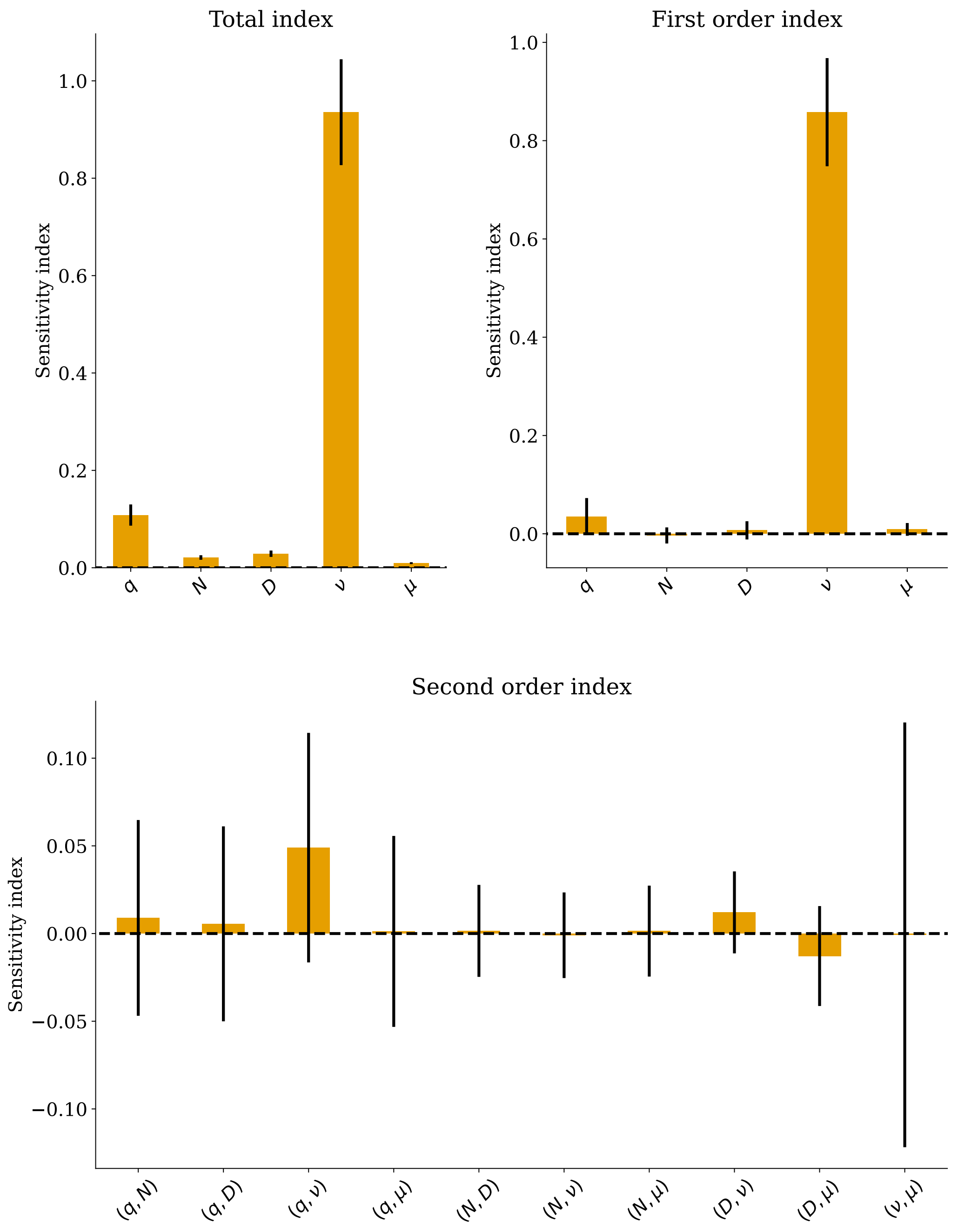

FIGURE H Sensitivity analysis of the number of plant species alive. Sobol sensitivity analysis of the number of plant species alive depending on five parameters: resource congestion q, nestedness N, connectance D, adaptation strength ν, and migration rate μ. The sample size per parameter was 512. The adaptation strength ν had the strongest effect on the variance of the outcome of the model.

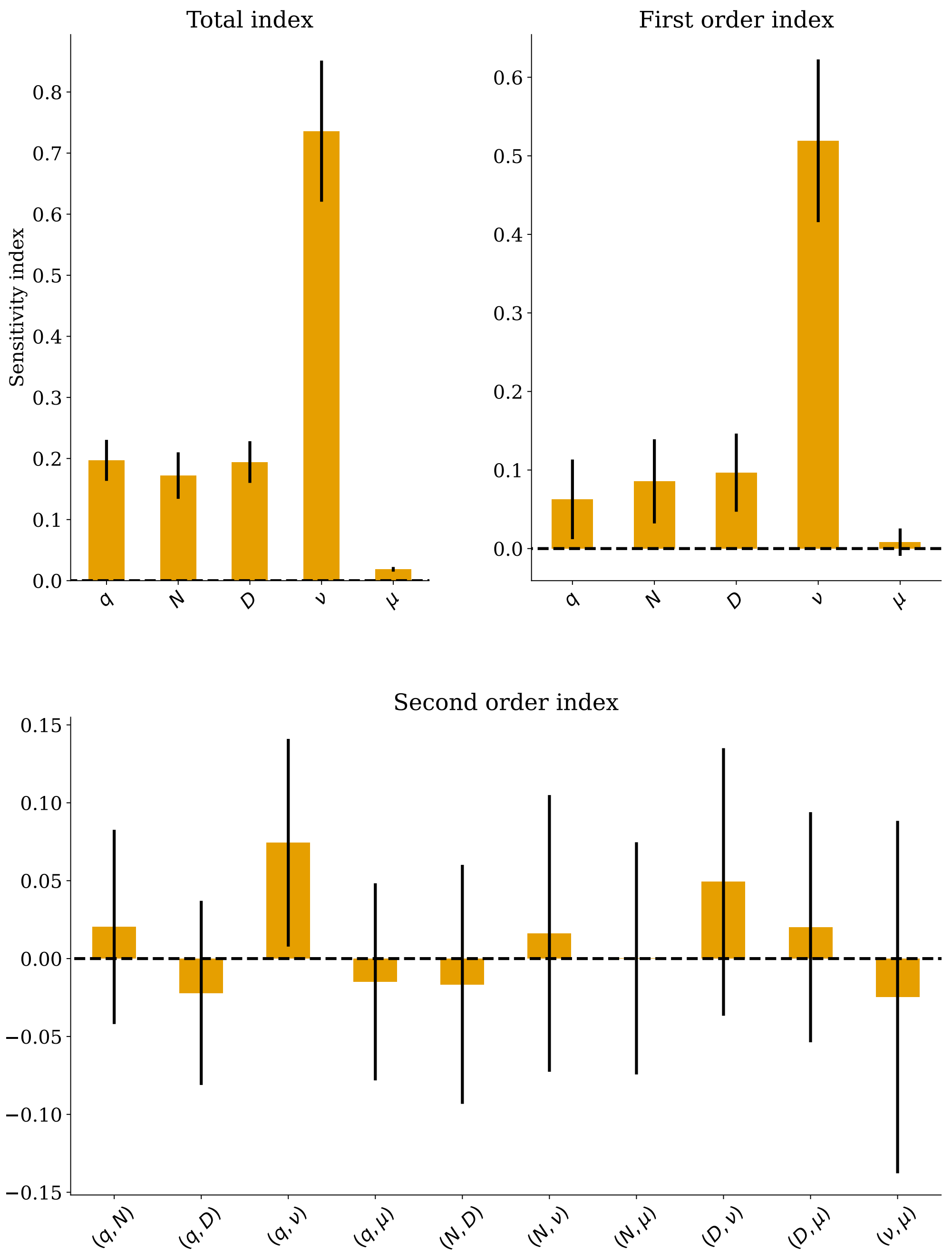

FIGURE 1 Sensitivity analysis of the number of pollinator species alive. Sobol sensitivity analysis of the number of pollinator species alive depending on five parameters: resource congestion q, nestedness N, connectance D, adaptation strength ν, and migration rate μ. The sample size per parameter was 512. The adaptation strength ν had the strongest effect on the variance of the number of pollinators alive. The migration rate μ only has a marginal effect.

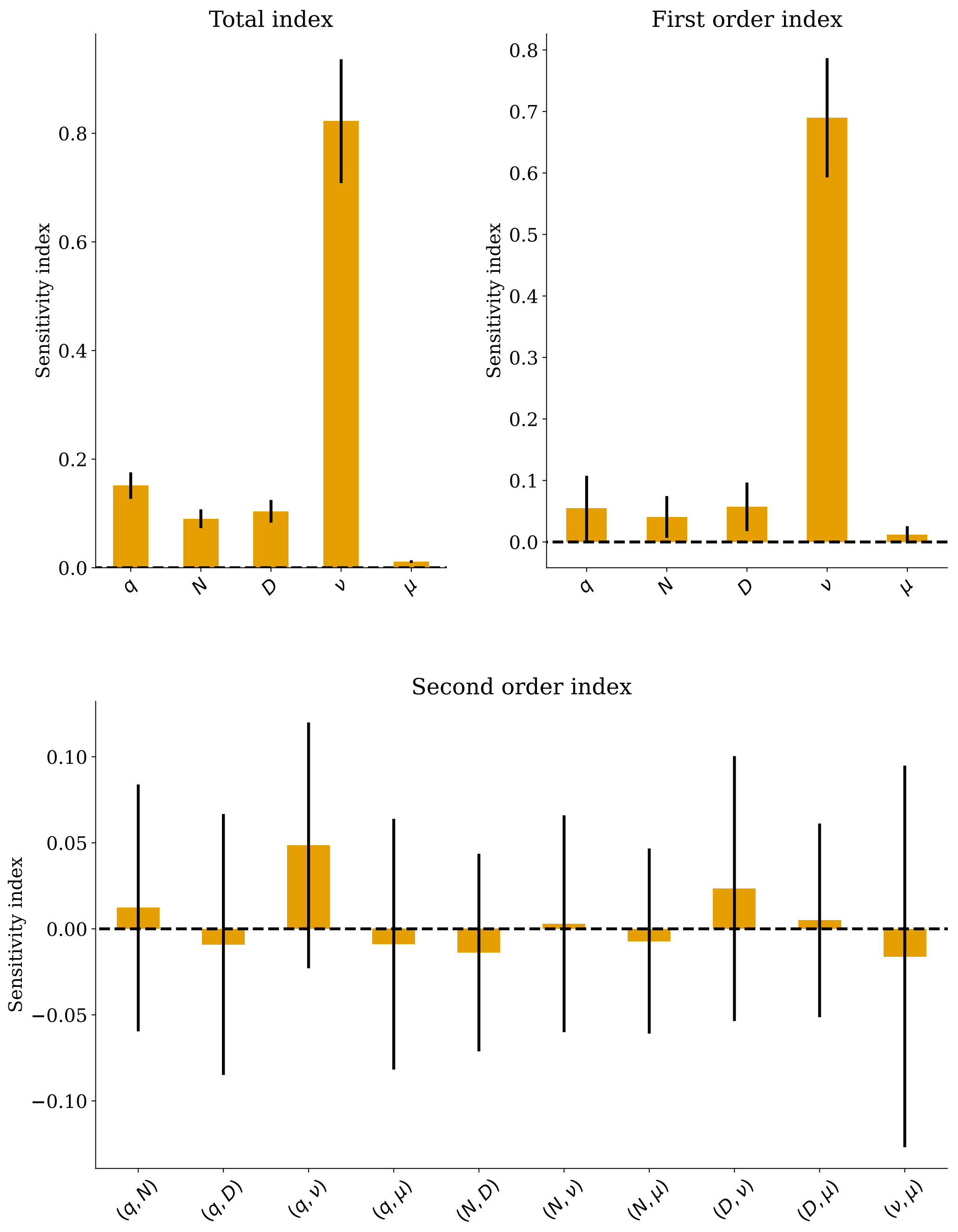

FIGURE J Sensitivity analysis of the total number of species alive. Sobol sensitivity analysis of the total number of species alive depending on five parameters: resource congestion q, nestedness N, connectance D, adaptation strength ν, and migration rate μ. The sample size per parameter was 512. The adaptation strength ν had the strongest effect on the variance of the outcome of the model.

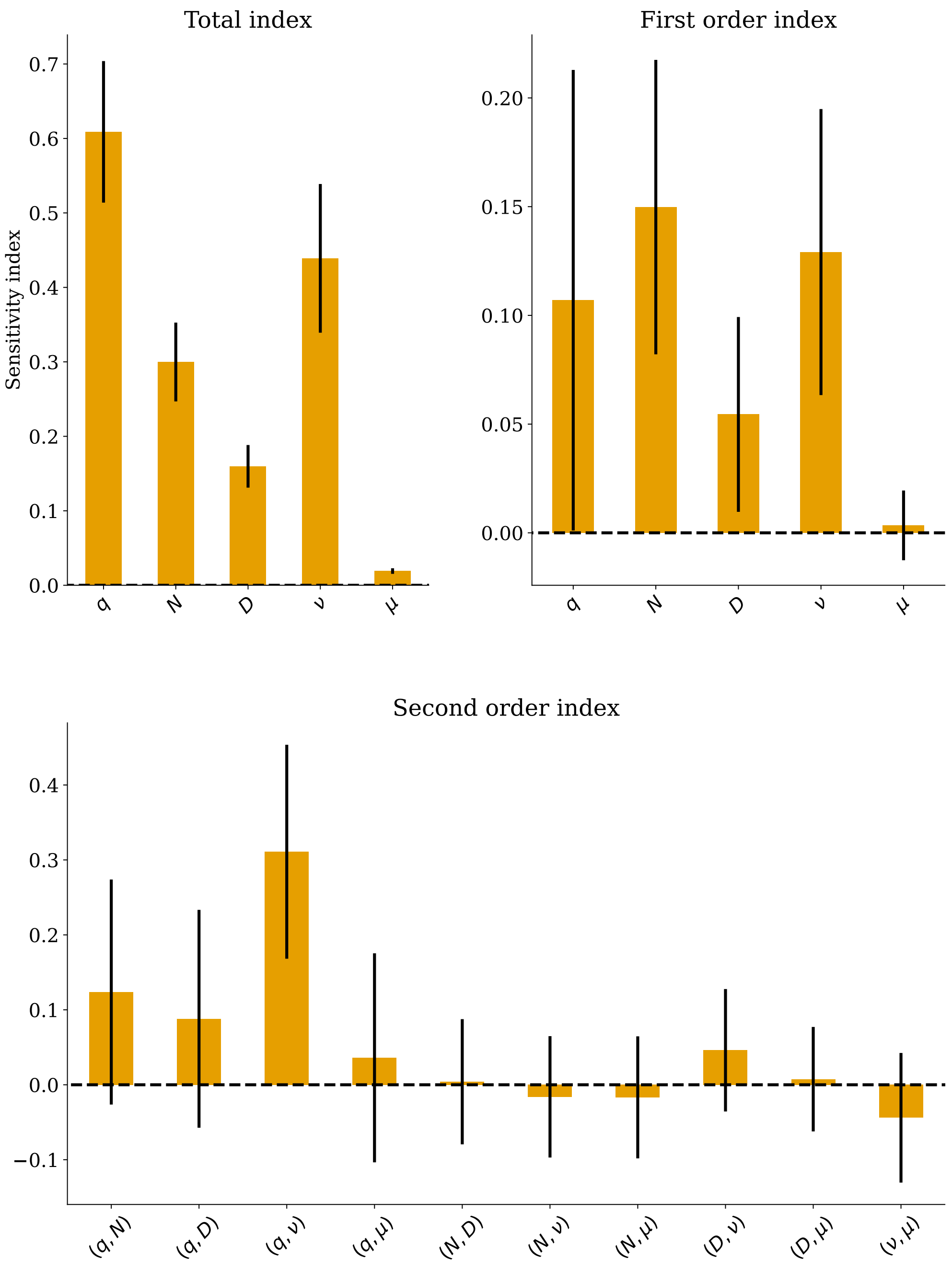

FIGURE K **Sensitivity analysis of the abundance of plant species.** Sobol sensitivity analysis of the average plant abundance depending on five parameters: resource congestion q, nestedness N, connectance D, adaptation strength ν, and migration rate μ. The sample size per parameter was 512.

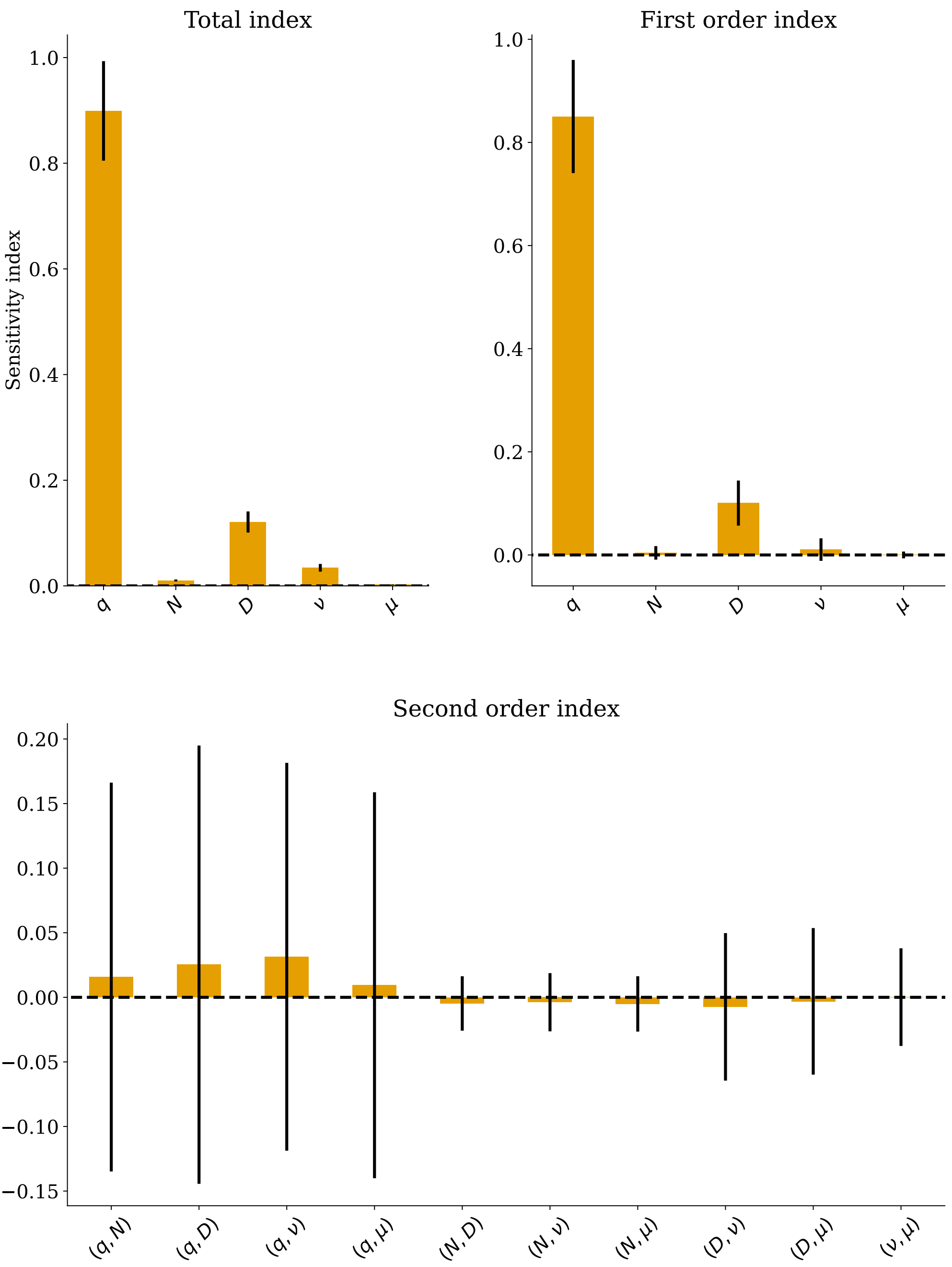

FIGURE L Sensitivity analysis of the abundance of pollinator species. Sobol sensitivity analysis of the average pollinator abundance depending on five parameters: resource congestion q, nestedness N, connectance D, adaptation strength ν, and migration rate μ. The sample size per parameter was 512.

| Sensitivity analysis on the critical driver of decline

Figure SM shows the sensitivity analysis on critical value of the driver of decline at which all pollinators species collapse (i.e., go extinct) d_A^{collapse} under the influence of varying the rate of change λ of the driver of decline d_A. Per definition, each result is only significant if the confidence interval does not include the sensitivity index of zero.

The sensitivity analysis was performed over six parameters, see Table SC. The number of samples per parameter was 512. In total, this led to a total Saltelli sample size of 7168. The simulation was run ten times for each sample, each time with a different network. Due to computational constraints, the networks were not tested on feasibility during these simulations. Therefore, the results might not properly represent the model, since infeasible networks might react different to external stressors and the rate of change.

TABLE C Parameters and their value ranges used for the sensitivity analysis on the critical driver of decline of collapse d_A^{collapse}. The fixed parameters can be found in Table SA. AF = Adaptive Foraging, d_A = driver of decline.

Parameter	Range of values	Explanation
q	$[0, 1]$	resource congestion
N	$[0.2, 0.7]$	nestedness
D	$[0.15, 0.35]$	connectance
ν	$[0, 1]$	AF strength
S^{init}	$[0, 4]$	initial abundance per species
λ	$[0.00001, 1]$	rate of change of d_A

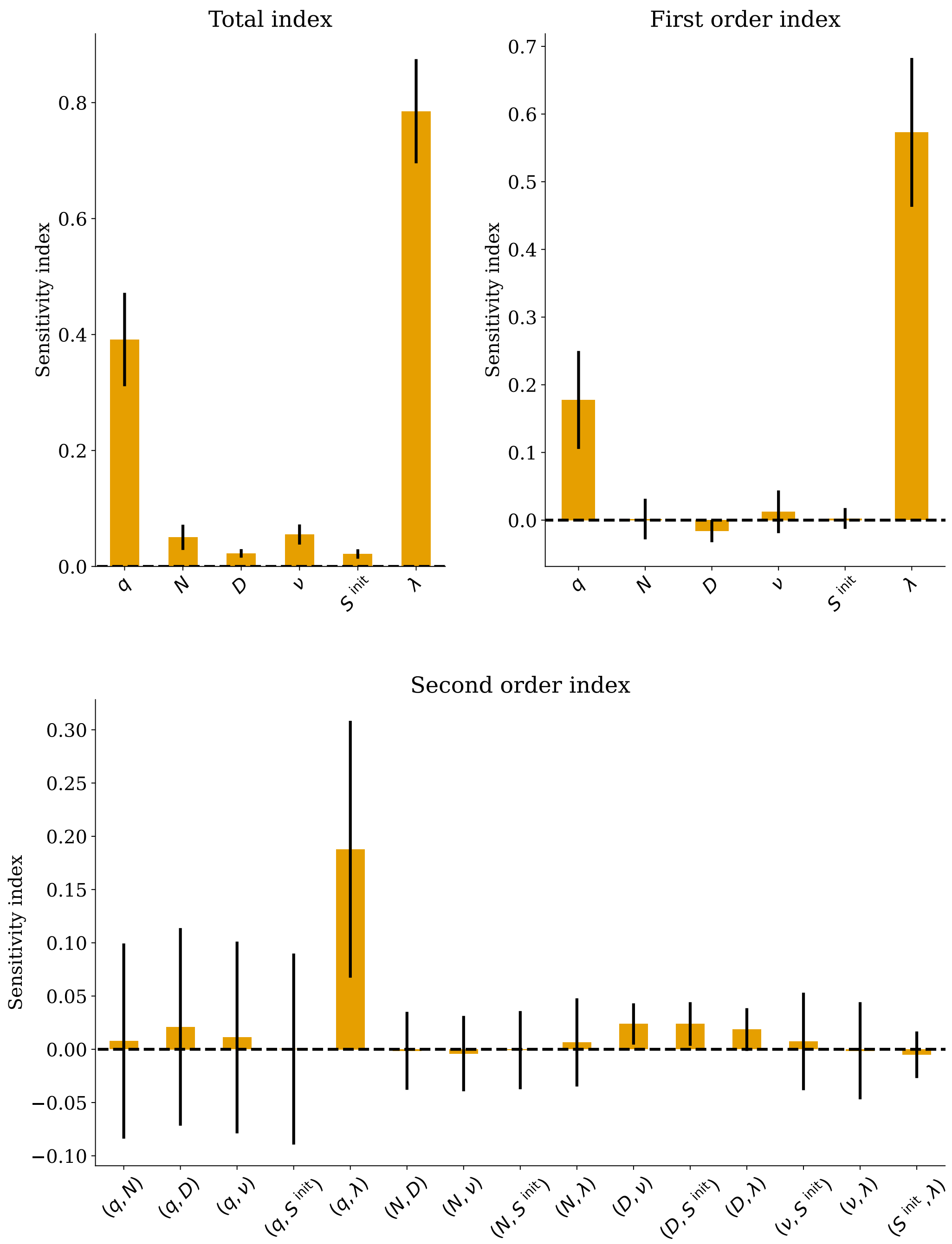

FIGURE M **Sensitivity analysis on the driver of decline d_A.** Sobol sensitivity analysis on the value of driver of decline at which all pollinator are extinct d_A^{collapse}, depending on six parameters: resource congestion q, nestedness N, connectance D, adaptation strength ν, initial abundance per species $S^{\text{ init}}$, and migration rate μ. The sample size per parameter was 512.

References

Bastolla, U, MA Fortuna, A Pascual-García, A Ferrera, B Luque, and J Bascompte (2009). "The architecture of mutualistic networks minimizes competition and increases biodiversity". *Nature* 458, pp. 1018–1020. doi: 10.1038/nature07950.

Hagberg, AA, PJ Swart, and DA Schult (2008). "Exploring network structure, dynamics, and function using NetworkX". *Proceedings of the 7th Python in Science Conference (SciPy2008)*, pp. 11–15.

Herman, J and W Usher (2017). "SALib: An open-source Python library for Sensitivity Analysis". *The Journal of Open Source Software* 2, p. 97. doi: 10.21105/joss.00097.

Lever, JJ, EH van Nes, M Scheffer, and J Bascompte (2014). "The sudden collapse of pollinator communities". *Ecology Letters* 17, pp. 350–359. doi: 10.1111/ele.12236.

Olesen, JM, J Bascompte, YL Dupont, H Elberling, C Rasmussen, and P Jordano (2011). "Missing and forbidden links in mutualistic networks". *Proceedings of the Royal Society B: Biological Sciences* 278, pp. 725–732. doi: 10.1098/rspb.2010.1371.

Saltelli, A (2002). "Making best use of model evaluations to compute sensitivity indices". *Computer Physics Communications* 145, pp. 280–297. doi: 10.1016/S0010-4655(02)00280-1.

Saltelli, A, P Annoni, I Azzini, F Campolongo, M Ratto, and S Tarantola (2010). "Variance based sensitivity analysis of model output. Design and estimator for the total sensitivity index". *Computer Physics Communications* 181, pp. 259–270. doi: 10.1016/j.cpc.2009.09.018.

Sobol, IM (2001). "Global sensitivity indices for nonlinear mathematical models and their Monte Carlo estimates". *Mathematics and Computers in Simulation* 55, pp. 271–280. doi: 10.1016/S0378-4754(00)00270-6.
