# Supplementary material for: Adaptive foraging of pollinators fosters gradual tipping under resource competition and rapid environmental change
Source: PLoS Comput Biol. 2024 Jan 9;20(1):e1011762. doi: 10.1371/journal.pcbi.1011762 (PMC10802948; doi:10.1371/journal.pcbi.1011762)
Supplement: Attachment — Submitted filename: pollinator_communities.pdf [file pcbi.1011762.s002.pdf]

"Early irreversible collapse of perturbed pollinator communities under rapid environmental change"

The present work concerns the dynamics and resilience of plant-pollinator communities subject to environmental stresses. The authors consider a nested network model of such a community and illustrate that rate-induced tipping can occur in multistable parameter regimes upon sufficiently fast variation of the external stressor process (parameter). Networks connections are sampled randomly and only feasible networks are retained for further study, i.e. those for which the system allows coexistence of all species with zero environmental stress. They extend their modeling framework to allow adaptive foraging strategies by giving dynamics to the foraging weights (assumed fixed constants in previous studies). Rate-induced tipping persists in this framework and shows some qualitatively different behavior to the constant foraging case.

The paper is well-written, considers a timely topic and is likely to be of interest to a substantial readership. I enjoyed the paper but I think the authors perhaps put too much emphasis on the finding of rate-induced tipping and not enough emphasis on the most novel aspect of the analysis, the addition of adaptive foraging. I have some comments and questions for the authors below that I would like to see addressed but this is an interesting piece of work and could be acceptable with some revisions.

Comments/questions:

- Line 8: "enhances" is somewhat ambiguous to me here. This is a crucial point for the reader to understand without reading the paper so I would reword this sentence to make the point more definitively, especially since the impact of adaptive foraging is a crucial novelty of the work.
- Bistability:
  (1) Is a given parameter regime called bistable if it has two stable equilibria for at least one feasible network? This might be a misunderstanding on my part but, if this is the meaning, it could be clarified further in the text.
  (2) How do the authors identify the bistable regime(s)?
  (3) Are there ever more than two stable equilibria? I haven't checked the stability thoroughly but there are families of boundary equilibria so it seems plausible there could be more than two stable equilibria for a given network, especially if we look over 100 different networks.
- Figures 2 and 3:
  (1) Can the authors comment on the apparent non-monotonicity of the orange (and green?) curve(s) in Fig. 3A for very small λ?
  (2) For the orange curve in Fig. 2A, the shaded region should indicate the first to third quartile ranges according to the caption. Does this indicate that the persistence distribution goes from skewed in one direction, to more symmetric, to skewed in the other as λ increases? Are there histograms of these distributions for say $\lambda = 0.15$ and $\lambda = 0.4$? If so, it seems that they would be qualitatively different.
  (3) The same question as above could also be posed of Fig. 3 A. In this case I'm also wondering why the variance of the green curve is so large and in particular, so large in relation to the variance of the orange curve? It seems there are some interesting non-monotonic changes in the variance going on here as θ varies.
- General point on novelty: The abstract does not clearly articulate the impact of including adaptation and the author summary does not mention the impact of adaptation on

outcomes at all. To me, the finding of rate-induced tipping in this system seems relatively unsurprising but the analysis of the impact of adapative foraging, and the fact that it exacerbates undesirable tipping events, is most interesting. Hence I think this is a crucial point to emphasize.

Minor comments/corrections:

- Definitions: I couldn't easily find a clear definition of "bifurcation-induced tipping" in the main text; this could be added to the introduction. Lines 47-48 purport to define a "tipping point" but I think instead define a tipping event (the tipping point would the threshold, rather than the system trajectory that results from crossing the threshold). Line 113 uses the term "critical value" but it might be better to note that this is a tipping point or threshold to keep terminology consistent.
- Line 298: The authors say they run simulations from the "extinct state" but if a species is identically at zero abundance, it seems that it can't recover? Are the authors using the fact that the abundances are starting from a value above numerical zero (which might cause numerical issues) or is the algorithm working differently than my reading suggests?
- I appreciate that the authors carried out a thorough sensitivity analysis in the supplement; it might be worth a sentence somewhere in the main text to point the interested reader to it.
