# Supplementary material for: Adaptive foraging of pollinators fosters gradual tipping under resource competition and rapid environmental change
Source: PLoS Comput Biol. 2024 Jan 9;20(1):e1011762. doi: 10.1371/journal.pcbi.1011762 (PMC10802948; doi:10.1371/journal.pcbi.1011762)
Supplement: Attachment — Submitted filename: Referee reply.pdf [file pcbi.1011762.s003.pdf]

Editors' comments:

Thank you very much for submitting your manuscript "Early irreversible collapse of perturbed pollinator communities under rapid environmental change" for consideration at PLOS Computational Biology.

As with all papers reviewed by the journal, your manuscript was reviewed by members of the editorial board and by independent reviewers. In light of the split reviews (below this email), we cannot accept the manuscript in its current form but we would like to invite the resubmission of a significantly-revised version that takes into account the reviewers' comments, in particular those of reviewer 1 considering the writing, motivation and implications of the work.
Please consider the question of whether a rate-induced tipping point is to be expected given the existence of bi-stability, and the recommendation that the novelty of the work resides on the consideration of adaptive foraging. Also, reviewer 1 raises the question of any empirical evidence for community collapse in these kinds of systems that would motivate the focus of the study. If that evidence is not there, we would recommend a clear emphasis on the theory, especially on the focus on adaptive foraging, and the more clear presentation of the relevance of this kind of transition vs. the dynamical properties expected from bi-stability and an external driver.

We appreciate the opportunity to resubmit a significantly revised version of the manuscript originally titled "Early irreversible collapse of perturbed pollinator communities under rapid environmental change."
We thank the editor and referees for their constructive feedback. It helped to shape the focus of the paper. Following the suggestions, we have substantially revised the writing, motivation, and implications of the work, and we added different analyses in order to address the points raised. Namely, we have focused the core of the revision on four main points: i) refocusing the research around adaptive foraging, ii) addressing the limits to the practical implications given the lack of experimental evidence supporting community-level collapse, iii) extending the analysis of the model and rewriting the results to incorporate the newly added figures, and iv) adding extra discussion to better incorporate the implications of rate-induced tipping and effect of adaptive foraging on the tipping dynamics of pollinator communities.

Briefly, we agree that an important innovative point of the paper is contributing a model of pollinator communities with adaptation. We have substantially changed the text to make this a bigger part of the focus of the paper, including adding an additional figure featuring the process of adaptive foraging (Figure 5).
We believe that the analysis of rate-induced transitions is still important since, beyond adding an important stressor to existing models, it is also the research question around rate-induced tipping that creates the need for considering an adaptive community. Furthermore, we are not aware of a study showing that rate-induced transitions are generally present. We agree that for a traditional 1D normal-form saddle-node bifurcation or the cusp bifurcation, in the presence of perturbations or noise, this could be proved, but we are not aware of such a paper. Further, pollinator communities are a multidimensional system and adaptive capacity further increases its dimensionality, making it hard to prove such general statements.
Finally, we agree with the reviewer and editors about the implications for conservation, and we were more careful (and accurate) in this revised version. To address this we

focused on pointing out how this work extends previous theoretical studies and note that empirical validation is missing.

As per the request of one of the referees, and for clarity, we have also rearranged the two previous methods sections into a single Model section and a Methods section with the details of the calculations. If this is too unconventional, we can create a merged “Model and Methods” section, without any additional change.

Below we provide a more detailed description of how we address these points as well as all remaining points raised by the referees.

We hope to have satisfactorily addressed all the points and, in doing so, have produced a manuscript relevant to the audience of PLOS Computational Biology.

Reviewer 1

This is a theoretical paper, which builds on previous studies (in particular Lever et al. 2014) investigating the emergence of community scale tipping points in a plant-pollinator model. Here, the authors add adaptive foraging of the pollinators in the model (i.e. pollinators can adapt their relative investments in their plant partners through time) and ask whether this mechanism affects the probability of community-scale tipping. The authors are in particular interested in rate-induced tipping: in the simulations, the driver of degradation (which is a linear loss term for the pollinators) can either be fixed or change in time, which allows investigating how the relative time scales of the processes affect tipping. One of the original aspects of the study is that the foraging effort is itself considered to be a dynamical variable.
I found the study interesting from a theoretical point of view. However, the justification of the study (the main objective and novelty of the paper) and the interpretation of the results are very misleading.

We appreciate the recognition of the original aspects of the paper, and we took your advice in rephrasing the focus of the paper. See how we addressed each point below.

The way things are presented gives the impression that the study is based on strong empirical support, while it's not. This is an extension of a previous modeling study (Lever et al. 2014), which was itself not based on any empirical studies. As far as I know, there hasn't been a single observation of a community-scale tipping point in a plant-pollinator community. As mentioned in Latty 2019 (cited in the paper): « We found only 1 empirical example of a tipping point or threshold response to stress at the colony level (Bryden et al 2013), and no empirical examples at the population or community level." Also, it's noteworthy that, even in models, community-scale tipping points are an exception rather than the rule. It only happens for certain network structures (and very specific dynamical equations). Therefore, it's important to realize that the setting of the study is a very particular one. This does not mean that this work is not interesting, but this means that the justification of the study must be rewritten to justify why it's relevant to investigate the emergence of community-scale tipping points in a plant-pollinator community model. Also - and maybe even more importantly - the references to applications of this work for conservation must be presented much more carefully.

This is an important point that we have addressed in the revision. The evidence for community-scale collapse is limited, in particular, given its lack of precise and universal definition. Indeed, our work does support the idea that community-scale collapse is rare, in the sense that the community is unlikely to disappear all at the same time point. This analysis is possible in our article because, as opposed to previous studies that looked into static external environments, the two timescales (that of the external environment and that of the pollinators) are coupled, and what we observe is a sequence of "failures" (in case of adaptive foraging) before the whole community disappears. Adaptive foraging strengthens this effect, with some species abundances even seeing a temporary boost. We have now stressed this point to a larger extent, including in the abstract. Some examples are below.

We explicitly changed the paragraph in the introduction to include the lack of empirical evidence (underlined text added):

“There is no direct empirical evidence for tipping in pollinator communities \cite{Latty2019}. In their review, \cite{Latty2019} found just one example of tipping in a bee colony \cite{Latty2019, Bryden2013}. However, most of the theoretical work on pollinator communities has found evidence for the potential for tipping points \citep{Latty2019, Dakos2014, Lever2014, Meng2021, Jiang2019, Huang2021}, especially those based on Lotka-Volterra type of dynamics.”

We also stressed it again in the discussion:
“The evidence for community-scale collapse draws mainly from theoretical work, without direct empirical observations of such an event \citep{Latty2019}. Indeed, our work supports the idea that community-scale collapse---in its traditional narrow sense---is rare because pollinator communities are unlikely to disappear at the same time. This analysis was possible with our model because of two reasons. First, as opposed to previous studies that looked into static external environments, the two timescales (that of the external environment and that of the pollinators) are coupled. Second, by including adaptive foraging, we include part of the natural adaptive capacity of pollinator communities to offset environmental change. What we observe in our model with adaptive foraging is a sequence of "failures"---tipping events of single or small groups of pollinator species---before the whole community disappears. Some species abundances---mainly generalist species---even see a temporary boost after the extinction of a few species due to the decrease in competition over resources. The fact that specialist species are the first to disappear seems to align with empirical studies \cite{Latty2019, aizen2012specialization, Burkle2013}.”

We have also revised our implications in terms of conservation. In this version, we mention our insights as contributing to the works on resilience (e.g., by Bascompte and Scheffer, “The Resilience of Plant–Pollinator Networks”, 2023), which is a more indirect contribution to conservation. Here is an example,

“Finally, our study also has implications for the study and notions of resilience. Since Holling \cite{Holling1973}, the concept has evolved from local bounce-back speed metrics to the ability of systems to reorganize under change and find new (but productive) equilibria. In the context of pollinator communities, these concepts are useful to define resilience in terms of the region of external conditions for plants and pollinators that allow the system to perform such a transformation \cite{bascompte2023resilience}. However, these external conditions cannot be seen (quasi-)statically. New uses of resilience must be sure to incorporate rapid external change.”

We have also mentioned that it is not just our work alone that contributes to better management practices, but the work on rate-induced transitions in general. This is the paragraph we identified with the strongest statement in the previous version, which we rewrote.

“The management of ecosystems, so far, has been concerned with establishing the limits to the abundance of species and limits to the external environmental conditions of these ecosystems. For instance, the group on the post-2020 Global Biodiversity Framework mentions the goal of a "reduction of ecological footprint in an equitable manner (...) within planetary boundaries by 2050" \citep{GDF_post2020}; other works advocate the use of fixed targets as opposed to net rates \citep{buschke2020reduced}. The work on rate-induced transitions, to which our work contributes shows that to determine targets of safe operation, it is important to consider not only the extent of change in external conditions but also the rate of change in these. Only then can we make a correct assessment of the resilience and recovery of these ecosystems. By neglecting that external conditions change over time, alterations in dynamics due to the rate of change are overlooked \citep{Feudel2018}. In practice, the rate of change can be a boundary in itself.”

Furthermore, the novelty of the work is not really clear in this version of the text. The way the text reads suggests that the novelty is that rate-induced tipping is found in the model. However, rate-induced tipping can happen in any dynamical system with bistability. So, there is no surprise that this can happen in this specific model too.

We agree that the focus should be changed, and we did. We believe the text is much improved and of broader interest to the readership of PLoS CB with this new focus.

However, as far as we are aware, there exists no proof that bistable systems always have (or don’t have) rate-induced tipping, so we believe it is still an important concept to understand systems’ resilience. Only recently studies in other systems have found these types of transitions (e.g., Siteur with salt marshes and Vanselow in predator-prey systems), but mutualistic communities have not been explored.
Contrary to these previous studies on rate-induced tipping, here, we study it in the context of networked and high-dimensionality systems, where general statements are far from being established.

Beyond its originality, we strongly believe rate-induced tipping is important in terms of the conservation of ecosystems and pollinator communities since they give an extra constraint on the concept of safe or resilient environments and include time explicitly in environmental change. This is especially relevant since real environments are dynamic (and directional) and, therefore, rate-induced tipping adds to the concept of structural resilience, since it is not just the “safe” parameter range that is important but also how parameters change in that range that gives a measure of systems’ ability to recover. Therefore, there is a need to include rate dependencies and adaptivity to the models describing these systems.

To add substantiation to the importance and nuance of RIT, in unperturbed pollinator communities (in the classical sense of equilibrium), we do not find any rate-induced tipping. Only in perturbed communities do we find it. We moved Figure S6 from the SI to the main text as Figure 4 to demonstrate this more clearly and well as to make the results clearer.

We also hope that the rewriting of the text does a better job of highlighting the importance of rate-induced tipping points in systems' resilience, especially now that it is guided by the main context of adaptive foraging.

To me, the novelty resides in the introduction of adaptive foraging in a plant-pollinator model which has been shown to exhibit tipping point and to investigate the effect of this mechanism on the likelihood of tipping. I would rewrite the introduction with a much stronger focus on the foraging mechanism (why introducing this mechanism rather than another one, what are the evidence that it is particularly relevant from plant-pollinator systems, why looking at the links between foraging and tipping, etc).

We rewrote the abstract, introduction, and discussion to emphasize adaptive foraging. We also added additional results that compare adaptive and non-adaptive dynamics side-by-side. Importantly, we also changed the title, making sure the focus on the new mechanisms is there from the beginning.

I would therefore also expect to see more results about the effect of adaptive foraging. For example, how does introducing adaptive foraging affects the bifurcation diagram of the model, has the location of tipping changed when adaptive foraging is included? The size of hysteresis? The height of the collapse…?

This is a good suggestion that we directly followed.

We added an extra figure (figure 5) in the new manuscript featuring a representative simulation to show the process of adaptive foraging. This figure shows how the foraging effort of pollinator species evolves over time under increasing environmental deterioration, representing the time dynamics of the components that come directly out of the model. We hope this provides more insight into the new part of the model, the new dynamics, and also helps with supporting the other results.

We generated an extra figure (figure 6) showing how including adaptive foraging changes the bifurcation diagram of the average pollinator abundance. In general, adaptive foraging makes the collapse of pollinator communities less abrupt---something we have highlighted from the abstract to the discussion.

The new text more clearly refers to Figures S2-S5, which show how the bifurcations are affected by varying adaptation strengths and resource congestion. Namely,

In the new Figure 6, *"Adaptation changes the bifurcation diagram. The collapse is less abrupt for pollinator communities with adaptive foraging. Resource competition decreases the size of the bistable area if there is no adaptive foraging. Supporting Information Figures S2 - S5 show the full bifurcation diagrams of all individual pollinator species for different settings of v and q."*

In the text, these results are useful again for the dynamics at species level:

“At the same time, both the collapse and recovery of pollinators become less abrupt. Looking at individual species, as illustrated in Supporting Information Figures S2-S5, some high-degree and highly abundant species can survive longer after the partial collapse of the system.”

The methods are currently split in two parts but I would merge them. In particular, the equations are needed to understand the results. Also, the way adaptive foraging is modeled needs to be more explained (make figures, show some dynamics, give an intuition of how alpha varies depending on the relative abundance of species by building a few toy examples). Sharing the code would have greatly helped.

We have merged and reworked the two parts. We now have a MODEL section with the different parts of the model described and including all equations. Then we have a METHODS section on how we analyze specific parts of the model.

We made numerous (small) edits to clarify the text. Most substantially, we rewrote the first part of the original model description to avoid stating things twice. We also added how we initialized the foraging effort alpha, which was missing in the previous draft, to the end of the paragraph where we introduce the foraging effort alpha: *“The foraging effort α is initialised such that the initial foraging effort is evenly distributed over all connected plant species.”*

Furthermore, we added some clarifications to the experiment descriptions at the end of the Methods section. We describe the rationale for choosing to include non-feasible networks in the analysis of bifurcation-induced tipping under varying resource congestion q.

The code was available via a link to Github through the Data Availability Statement: https://github.com/TerpstraS/pollcomm. We now made a static version available via Zenodo with the following link: https://doi.org/10.5281/zenodo.8268438. We added this explicitly to the manuscript text itself: “*The data and model are published online and can be accessed through the following link: https://doi.org/10.5281/zenodo.8268438*”

The new figure 5, which we mention above, also clarifies the dynamics of the components of the direct model, how the adaptive foraging works, and how it shapes the dynamics under deteriorating environments. The top row of the figure shows how the pollinator and plant abundances change over time. The other panels show for all the different pollinator species how their foraging effort changes over time.

I give more detailed comments below.

Detailed comments

l. 2-4: “Previous studies…”: specify “previous theoretical studies….” As far as I know, there is no empirical observation of such behavior

We agree and changed "*Previous studies*" to "*Previous theoretical studies*". We now do this consistently throughout the text.

l.5-7: "we show that…": the way things are formulated, it seems that the possibility for rate-induced tipping points is a primary result of your work. But this is well-known and expected in model where you have bistability. Maybe it's only a matter of formulation (things are written in terms that are too general lines 5-7), but I would try to more specifically highlight the novel results of the study, which as far as I understand are related to the effect of adaptive foraging on the dynamics of the model. These results are mentioned l. 8 ("additionally"), as something additional, whereas they should probably be the center of the abstract.

We have now changed the focus and these aspects are now roughly in the opposite order, with additional detail on model dynamics. We rephrased the abstract significantly to better reflect the novelty of our paper. The second half now reads:

"We model the adaptive foraging of pollinators in changing environments. Pollinator's adaptive foraging alters the dynamical responses of species, to the advantage of some---typically generalists---and the disadvantage of others, with systematic non-linear and non-monotonic effects on the abundance of particular species. We show that, in addition to the extent of environmental stress, the pace of change of environmental stress can also lead to the early collapse of both adaptive and nonadaptive pollinator communities. Specifically, perturbed communities exhibit rate-induced tipping points at stress levels within the safe boundary defined for constant stressors. With adaptive foraging, tipping is a more asynchronous collapse of species compared to nonadaptive pollinator communities, meaning that not all pollinator species reach a tipping event simultaneously. These results indicate that it is essential to consider the adaptive capacity of pollinator communities for monitoring and conservation. Both the extent and the rate of stress relative to the ability of communities to recover are critical environmental boundaries."

L. 9-11: Because of the various reasons explained in this text, I would delete l. 9-11 (from "Thus, ecosystem management…"

We have toned down this statement, for those reasons. We have not fully eliminated it since, as we said before, there are important concepts that need to be translated into resilience research, with real-world implications for monitoring. Therefore, we now write the following at the end of the abstract:

"*These results indicate that it is essential to consider the adaptive capacity of pollinator communities for monitoring and conservation. Both the extent and the rate of stress relative to the ability of communities to recover are critical environmental boundaries.*"

l. 13-18: In the same way as for the abstract, the author summary should highlight the novelty of the paper better. The way things are written, it seems that the results were already well-known in the literature.

We appreciate this. We have rewritten the authors' summary. We also included the influence of adaptive foraging on the model behaviour in the summary. E.g.,

"Our model shows that, with adaptation, the extinction of species is more sequential and spread out in time and may even temporarily favour especially generalist species."

l. 20: "pollinator communities, ecosystems of plant and pollinator species…": the formulation is incorrect. Communities are not ecosystems… Maybe just reformulate as "Communities of plants and pollinators…"?

Thanks for noticing and pointing it out. We rephrased it as follows:
"*Pollinator communities, which consist of interconnected plant and pollinator species, form a vital part of many ecosystems and enhance their biodiversity.*"

l. 60-61: the way things are written is clumsy. I agree that I don't know of a theoretical study on plant-pollinator networks that specifically looked at rate-induced tipping. However, the theoretical results on the topic are so generic that we know that this can happen in such system and we don't need to specifically test it here. What I find more interesting and novel here is the question of how adaptive foraging affect rate-induced tipping. I would try to reformulate this.

We have made extensive rewriting. We have now focused more clearly on the novel aspects of our study. Not just that rate-induced tipping is present here but, importantly and with novelty, we focus on how it happens in these networked and heterogeneous systems.
We also highlight how it has so far been ignored when defining safe operating spaces in this context and, thus, poses additional risks.

l. 78: A reference is needed at the end of the sentence "Adaptive networks…"

We agree with the reviewer. The Valdovinos et al. 2013 reference is also meant for this sentence. We rewrote the two sentences at lines 78 and 79 into one sentence with the Valdovinos et al. 2013 reference.
"Adaptive networks seem to improve the persistence of pollinator communities, and adaptive foraging sustains biodiversity in pollinator communities \citep{Valdovinos2013}."

l. 88: "to test these hypothesis": which ones? There is no explicit hypothesis formulated (as far as I could see).

We agree that there were no clear hypotheses stated. In the revision, we changed the wording there too to avoid confusion and clarify the research questions.

Now the sentence "*Figure 1A illustrates the causal mechanisms considered in the model used to test these hypotheses.*" reads "*Figure 1A illustrates the causal mechanisms considered in the model.*"

To make the goal of the paper more clear, we added studying adaptive foraging more clearly to the mentioned objective of studying rate-induced tipping in the last paragraph of the introduction. In the last paragraph of the introduction the research questions are stated explicitly:

"As environments keep changing, adaptation may play an increasingly important role. Therefore, including adaptation and dependencies on the rate of change of environmental stressors in current models of pollinator communities is necessary to investigate whether rate-induced tipping is present and how it happens. In this work, the questions are whether i) in adaptive pollinator communities, irreversible tipping can occur due to the speed of environmental change, not just due to the extent of environmental change, and ii) how adaptive foraging changes the tipping dynamics of the model. The focus is on adaptive mechanisms at the ecological timescale, not the evolutionary one, since the latter acts at longer timescales. We will use the term adaptation in this sense. We expand on previous models \citep{Lever2014} by adding adaptive foraging depending on resource competition and a rate dependency to the driver of decline."

l. 108: please move the equations and the rest of the methods here. It's currently impossible for the reader to understand the results without having gone through the more detailed method. In particular, I did not understand the type of model you studied, and more specifically, how adaptive foraging was introduced in the model from the text alone. This point needs to be explained in more details.

We have now merged, expanded, and rearranged the two previous methods into a Model section and a Methods section (see details in a comment above). We also created subsections so it is easy to pinpoint precise parts of the model.

L. 115: "when initial abundances are low": does this mean that you ran simulations with high vs low initial abundances? How did initial conditions affect the outcome of the results and where is that shown?

This is an important point that we now highlight more significantly in the main text. As mentioned in the second paragraph of the discussion (line 171-172 of the original draft), rate-induced tipping only occurs when the system is perturbed out of equilibrium. This is equivalent to starting with low initial abundance (below the upper stable equilibrium). To make this point clearer, we moved Figure S6 from the Supporting Information to the main text (now Figure 4) and discussed its results. This figure shows both the effect of initial abundance and adaptive foraging on rate-induced tipping. We also explicitly state now the values for low and high abundance that were not clear in the previous version.

Figure 4: Line 104, you wrote: "At q = 0, there is no resource competition and, thus, no adaptive foraging. For q > 0, there is adaptive foraging." So why don't we have the same results between the left and right panels for q=0?

Thanks for pointing this out. This was a mistake in the text, we removed "*and, thus, no adaptive foraging*". Parameter q only affects the resource competition and does not control whether we have adaptive foraging or not. The adaptive foraging is controlled by the ν parameter. When ν equals 1, there is no adaptive foraging. To clarify this better, we added "*Adaptive foraging decreases with increasing ν , and there is no adaptive foraging at ν = 1.*" to the methods section directly after equation 3. Only when q = 0 and ν = 1 the model is the same as described by Lever et al. 2014. To clarify this better, we added "*The model is the same as described by Lever et al. 2014 when setting q = 0 and ν = 1 (i.e., no adaptive foraging and no resource competition).*" to the methods section directly after equation 3.

I was confused at this point, but I think that's also because I did not find the methods very clear on how adaptive foraging was incorporated in the model and how its effect was investigated. The fact that the methods is currently split in two parts and that the code for the analyses was not shared with the reviewers (or maybe I missed it?) did not help.

We have merged the methods and now provide a link to the code. See the more detailed response in the comment above and the data availability statement here:

"Data Availability Statement
"*The data and model are published online and can be accessed through the following link: https://doi.org/10.5281/zenodo.8268438*"

l. 254: I did not understand how you decide whether a connection exists? How do you generate the skeleton of the network?

The full algorithm for creating the bipartite, nested networks can be found in the Supporting Information S2. Unfortunately, we did not mention it in the main text. We have now fixed this issue. We added a clear reference from the text to this in the Method section (and not all sections are mentioned in the main text). The skeleton of the network generated by this algorithm has a structure similar to real-life plant-pollinator networks. See below the added text to the manuscript:

"Whether two species can interact with each other (and, thus, whether the $\boldsymbol{\beta}$-value between two species is nonzero) is dictated by a bipartite network describing the connections. We generate these bipartite networks using an algorithm as described in Supporting Information S2. The algorithm takes as input the number of plant species, number of pollinator species, nestedness, fraction of forbidden links, and connectivity. These networks have a similar---nested---structure as real-life plant-pollinator networks \cite{Medan2007, Lever2014}."

In the equations after l. 258, I don’t understand what Kp and KA are: are they the average number of connections of plants and pollinators (if it’s “the number of connections a plant has”, as defined l. 256, then why doesn’t it have the index i or j of the concerned plant/pollinator species?

That was a mistake. K_P and K_A are indeed the number of connections a given plant or pollinator species has. We added an index i and j to the equations, respectively, as suggested.

Equations after l. 256: there is no \rho in equation 1 so it’s unclear exactly how to reconciliate those.

There was a bit of a confusion in the terms indeed. And, to be fair, the parenthesis notation for functional dependency should just be revised in the scientific community as a whole.

The mutualism-derived growth, H(**ρ**), is a function of **ρ**, where **ρ** is the overall mutualistic benefit, which is given as an argument (the summation) to the function H(**ρ**) in equation 1. We now have a whole section dedicated to computing these terms. The beginning of that section is:

“Mutualistic interactions with resource competition

*The mutualism-derived growth is given by a Holling type-II function, H(**ρ**), which ensures the saturation of the mutualistic relation as the overall mutualistic benefit, **ρ**, increases. Precisely, $H(**ρ**) \equiv **ρ**(1 + **ρ**/h)^{-1}$, with h as a horizontal asymptote with a different value for each species \citep{Holling1959}. The mutualistic benefits, **ρ**, are the sums passed to the H functions for pollinators and plants in equation \ref{eq:ODE_A}. They are different for pollinators and plants, so we call them $\boldsymbol{\rho}_j^A$ and $\boldsymbol{\rho}_j^P$, respectively.”*

Then we go on about how to build those sums.

Equation 3, between l. 277 and 278: this is a key element of the model which is not stressed at all in the text. It would be important to highlight this point more (and earlier in the text), so that the reader understands that’s one of the originality of the paper.

We believe the new version of the text is now strongly connected to this equation as well as to the novel part of the text. We have rewritten the introduction, discussion, added new results, and changed the title of the article to focus on this aspect. The new structure of the methods also helps to find these novel points. There is a section on “*Mutualistic interactions with resource competition”* and a section on *“Adaptive foraging”*. We also state in those sections that here is where we extended the previous models.

l. 286: please replace “ecosystems” by “communities” (you’re not modeling ecosystems here)

Thank you for pointing this out. We replaced “ecosystems” by “communities” as suggested.

l. 287: “…had a feasible solution,…” I found the formulation unclear: do you mean that had a feasible solution (even when some species may go extinct) or that had a solution such that all species initally presnet survive?

We mean that we had a solution where all species initially survived without external stressors (i.e., dA = 0). We write this explicitly now in the dedicated subsection of the Methods:

“Feasibility of networks
Different interactions among species generate different communities. To ensure that each mutualistic network had similar characteristics, we only investigated networks that had what we call a "feasible solution", meaning that all their species survive in equilibrium when there is no external stress ($d_A=0$) \citep{Lever2014}.”

l. 291-292: “can be seen as a measure of resilience”. Not of resilience (or according to which definition of resilience). You could say persistence? Or more generally ‘stability’?

This should indeed be “overall persistence of the networks”. We replaced resilience with persistence. The term stability in this paper also includes the state with zero abundances, which we want to explicitly exclude here, and might lead to confusion given the focus on bistability. We leave the term resilience for structural resilience in the Holling sense.

Reviewer 2

Please see the attached PDF for detailed comments. The paper is undoubtedly interesting and I recommend some minor revisions.

We appreciate the very positive assessment of our paper, and we have addressed the concerns in detail below. Following your comment and the suggestion of another reviewer, we have enhanced the focus on the adaptive foraging component of the model, while still maintaining the question of the impact of varying environmental stressors in these communities.

The present work concerns the dynamics and resilience of plant-pollinator communities subject to environmental stresses. The authors consider a nested network model of such a community and illustrate that rate-induced tipping can occur in multistable parameter regimes upon sufficiently fast variation of the external stressor process (parameter). Networks connections are sampled randomly and only feasible networks are retained for further study, i.e. those for which the system allows coexistence of all species with zero environmental stress. They extend their modeling framework to allow adaptive foraging strategies by giving dynamics to the foraging weights (assumed fixed constants in previous studies). Rate-induced tipping persists in this framework and shows some qualitatively different behavior to the constant foraging case.
The paper is well-written, considers a timely topic and is likely to be of interest to a substantial readership. I enjoyed the paper but I think the authors perhaps put too much emphasis on the finding of rate-induced tipping and not enough emphasis on the most novel aspect of the analysis, the addition of adaptive foraging. I have some comments and questions for the authors

below that I would like to see addressed but this is an interesting piece of work and could be acceptable with some revisions.

Comments/questions:

- Line 8: “enhances” is somewhat ambiguous to me here. This is a crucial point for the reader to understand without reading the paper so I would reword this sentence to make the point more definitively, especially since the impact of adaptive foraging is a crucial novelty of the work.

We significantly revised the abstract and authors summary. We now more explicitly mention the effect of adaptive foraging on rate-induced tipping. Namely, you can now read the following two sentences in the abstract:

“Pollinator's adaptive foraging alters the dynamical responses of species, to the advantage of some---typically generalists---and the disadvantage of others, with systematic non-linear and non-monotonic effects on the abundance of particular species.”
“With adaptive foraging, tipping is a more asynchronous collapse of species compared to nonadaptive pollinator communities, meaning that not all pollinator species reach a tipping event simultaneously. These results indicate that it is essential to consider the adaptive capacity of pollinator communities for monitoring and conservation. Both the extent and the rate of stress relative to the ability of communities to recover are critical environmental boundaries.”

And the sentence in the authors’ summary:

“Our model shows that, with adaptation, the extinction of species is more sequential and spread out in time and may even temporarily favour especially generalist species.”

- Bistability:

(1) Is a given parameter regime called bistable if it has two stable equilibria for at least one feasible network? This might be a misunderstanding on my part but, if this is the meaning, it could be clarified further in the text.

We were indeed loose in the way we used the terminology.

We now include the operational definition of bistability we used in the new Methods section:
“The region of bistability is the region between $d_A^{recovery}$ *and* $d_A^{collpase}$ *for each network, given* $d_A^{recovery} < d_A^{collpase}$*. The inequality condition guarantees that there are two stable equilibria for a given parameter set and network.”*

So the definition is per network. As you can see (including in the new figures), even including the variability in recovery and collapse points across networks (represented with the bands and bars over the points as the standard deviations across networks and not the error of the mean

over networks), there is still a region where all are bistable, so it is possible to also talk about regions of bistability for, if not all, at least most networks.
We also reviewed the whole text and made it more clear what bistability meant per case (either in a specific system with a specific network of interactions or at the level of the ensemble of networks). In the discussion, we recover this idea:

"In this paper, both rate-induced tipping and bistability are community concepts, showing how the critical values of the rate of change of driver of decline can differ between pollinator communities. However, when extended to the species scale, this region is different for each species, with different species collapsing under different conditions."

As a minor point, note also that this definition is not dependent on the underlying network being feasible or not since an unfeasible network can still have a bistable regime (unless the unfeasibility makes all species extinct). However, since we mostly focus on feasible networks this is not an important point.

(2) How do the authors identify the bistable regime(s)?

See detail in the previous answer. In short, we identify bistable regimes by calculating the point of collapse and the point of recovery of the system under constant environments (this is specified in the new Methods section, and it is similar to Lever et al. (2014)). The parameter values between the point of recovery and the point of collapse is the bistable regime.

(3) Are there ever more than two stable equilibria? I haven't checked the stability thoroughly but there are families of boundary equilibria so it seems plausible there could be more than two stable equilibria for a given network, especially if we look over 100 different networks.

As far as our explorations indicate, these networks seem to have only two stable equilibria. We do have to notice a few design choices that contribute to this. The first is that for the feasible networks we impose that all species are alive (does the absence of one versus other species is not possible). The second is that we follow a specific path to and from extinction, increasing and then decreasing the driver. Finally, an important point, which probably generated part of this question, is that we do have a low rate of migration, so the boundary equilibria disappear. We expand on this point below, where you ask the extinct state.

In any case, when starting the experiments with a range of different initial abundances covering substantial parts of the state-space, the original system seems to always end up in one of the two stable equilibria; we haven't found that the system settles in another equilibrium. Since this is in line with previous analysis (e.g., Lever et al. (2014)), we haven't included a specific analysis of the equilibrium state space. However, it is true that the exact values of the equilibria are different for each (randomly) generated network, but each network seems to exhibit two stable equilibria.
We show examples of the equilibria in Supporting Information Figures S2-S5 for different adaptation strengths and resource congestion values.

Since we focus on the path to full extinction, we do think the further exploration of multistability falls beyond this research. That said, it could be interesting to look into the possibility that one species goes extinct as opposed to the other depending on exactly where the system begins. We added a sentence about it in the discussion:

“Additionally, the analysis of possible different extinction pathways and consequent multi-stability could also shed light on the path dependency of these communities.”

- Figures 2 and 3:

(1) Can the authors comment on the apparent non-monotonicity of the orange (and green?) curve(s) in Fig. 3A for very small λ?

We had not reflected on the non-monotonicity since it was within the range of variation. To investigate this issue, we have now run the simulations with higher resolution in lambda (only for lower lambda, due to computational time) and increased the number of simulations to 500 instead of 100 (see the figure below). By doing so, we expected to decrease the error of the mean by a factor of sqrt(5)≈2.2 to understand whether the fluctuation was just due to variability or if there was an actual effect of the mean value of the simulations. Below, you can find the newly generated figure. The variation in the mean is now much reduced, hinting that it is not significant, and likely to disappear as we increase the number of simulations. We considered plotting the bands as the error of the mean to highlight these facts, as opposed to the quartiles of the simulations. However, we do think the point you make below about the distribution of the results is an important one, and given that that distribution is not unimodal, plotting error bands is even less informative (and possibly misleading) than quartile bands.

That said, non-monotonicity for individual networks can exist. However, this is less obvious, and seems not to be the case, for the averages over an ensemble of networks.

Since our results do not focus on the small rates of change, or this non-monotonicity in particular, we believe this can be left out of the text.

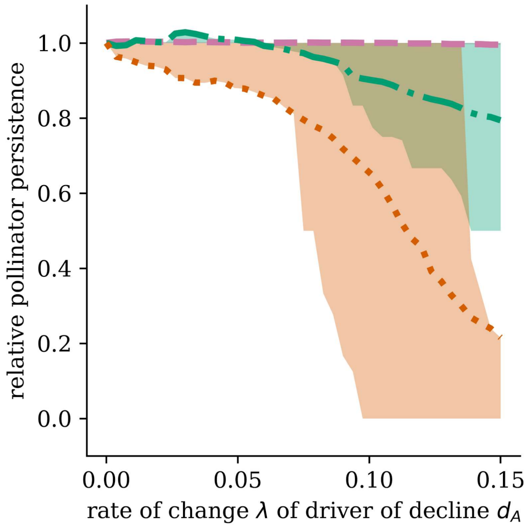

(2) For the orange curve in Fig. 2A, the shaded region should indicate the first to third quartile ranges according to the caption. Does this indicate that the persistence distribution goes from skewed in one direction, to more symmetric, to skewed in the other as λ increases? Are there histograms of these distributions for say λ = 0.15 and λ = 0.4? If so, it seems that they would be qualitatively different.

The distributions for both the nonadaptive and adaptive model are indeed skewed and change over the range of λ-values (see the two figures below which we added in the SI section 4 as Figures S6 and S7). Even more than being skewed, they are bimodal. At low λ, most networks contain all species with positive abundance with just a few extinct species in a subset of networks. At higher λ, some networks fully collapsed while some have still surviving species or are not yet fully collapsed. This leads to skewed distributions (in part necessary given that the relative pollinator persistence is bounded above 0). Some networks see a boost in relative pollinator persistence with persistence values higher than 1, but they are uncommon and occur only for, what we assume to be, very specific networks.
It could be an interesting point to explore which networks lead to that. We added a sentence at the end of the discussion about it.

"*In our model, a few specific networks saw an increase in relative pollinator persistence with environmental change. Systematically identifying the properties of those networks could also contribute to a better understanding of what structures improve pollinator communities resilience.*"

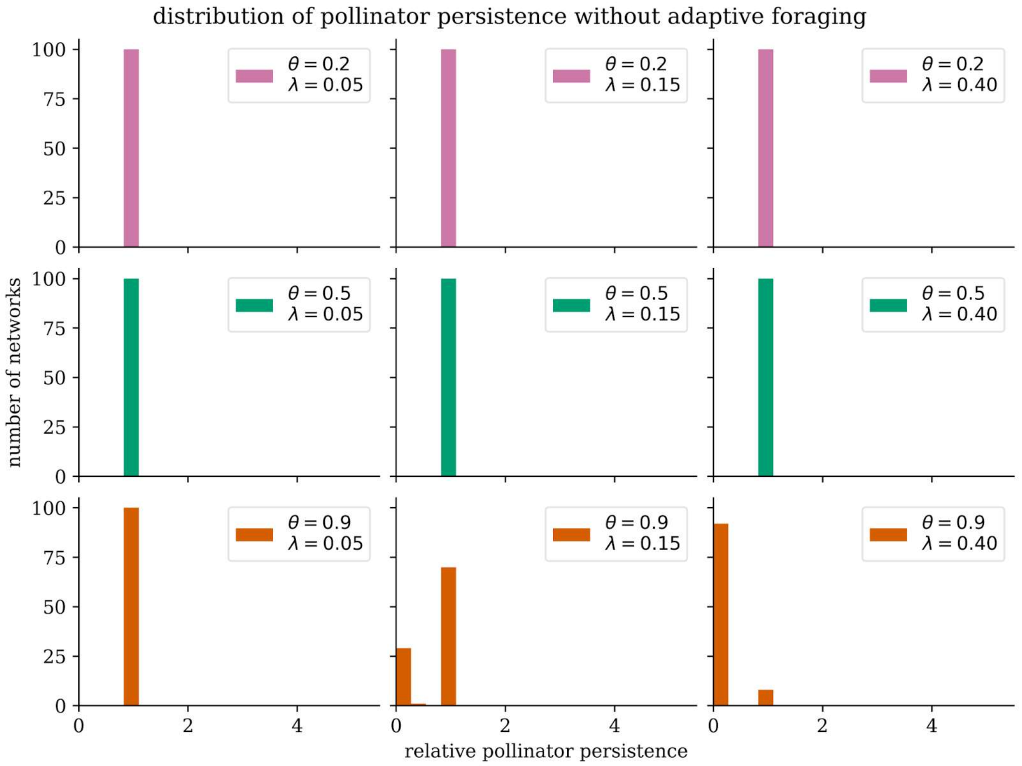

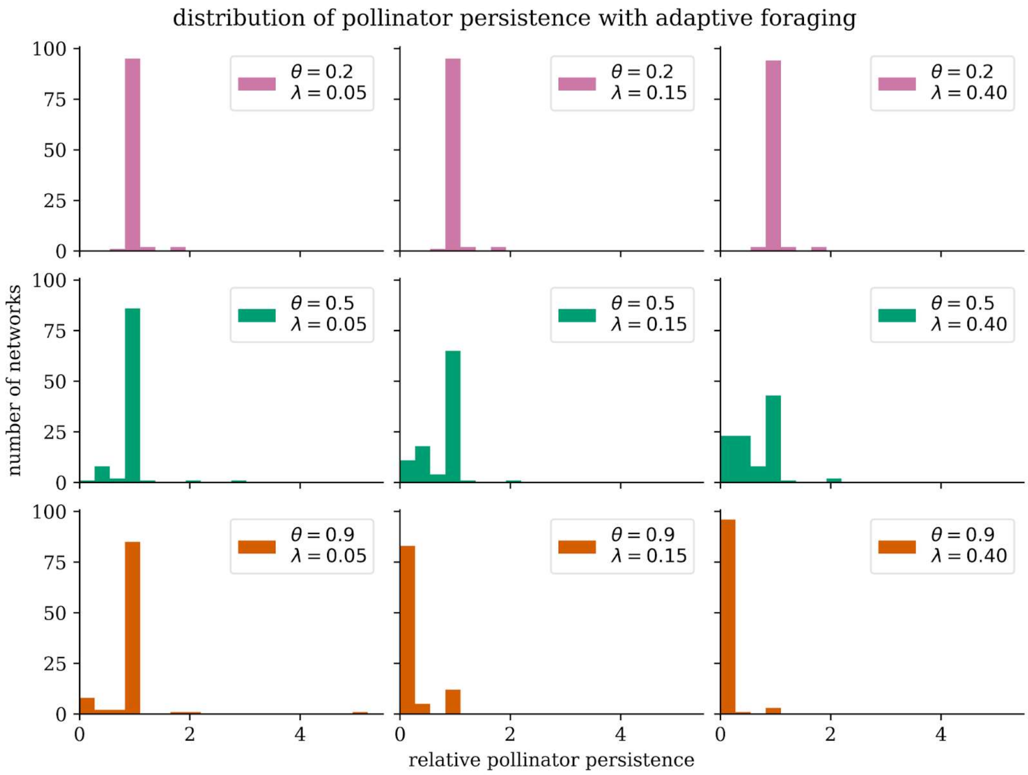

(3) The same question as above could also be posed of Fig. 3 A. In this case I'm also wondering why the variance of the green curve is so large and in particular, so large in relation to the variance of the orange curve? It seems there are some interesting non-monotonic changes in the variance going on here as θ varies.

This is an interesting point, and it relates exactly to the extinction process, and how it differs in the adaptive and non-adaptive cases.

In the adaptive case, we can see that the collapse of species is less abrupt than compared to the nonadaptive case. More specifically, fewer species go extinct simultaneously. This can be seen comparing how Figure 3B is less abrupt than Figure 2B. So, for the green curve, a lot of species are already collapsing (we chose theta to illustrate that) in the adaptive case, whereas the Figure 2A there were not.

We added a lot of additional focus on these differences between the adaptive and non-adaptive cases to the new text, as we mentioned above, as well as for the graduality in tipping for the adaptive communities. For instance, right after Figure 3, we write:

"This suggests that adaptive pollinator communities are more likely to collapse partially over a range of maximum drivers of decline, rather than exhibiting a complete collapse of all species at

a specific value of the driver. Later we show which species are affected throughout the gradual tipping process."

The new Figure 5 and the figures for tipping of individual species in SI (Figures S2-S5) are also illustrative of these dynamics. We have added the distribution of persistence to the SI (as Figures S6 and S7, see also our reply to the previous comment) all of which now also help convey this information.

- General point on novelty: The abstract does not clearly articulate the impact of including adaptation and the author summary does not mention the impact of adaptation on outcomes at all. To me, the finding of rate-induced tipping in this system seems relatively unsurprising but the analysis of the impact of adapative foraging, and the fact that it exacerbates undesirable tipping events, is most interesting. Hence I think this is a crucial point to emphasize.

As we mentioned above, we have taken your advice and significantly revised the abstract to clearly describe the novel aspects of our study with a bigger focus on adaptive foraging and the influence of adaptivity on rate-induced tipping.

We also rewrote the authors' summary and now include the impact of adaptation on outcomes.

We appreciate this observation and think the manuscript has greatly improved because of it.

Minor comments/corrections:

- Definitions: I couldn't easily find a clear definition of "bifurcation-induced tipping" in the main text; this could be added to the introduction. Lines 47-48 purport to define a "tipping point" but I think instead define a tipping event (the tipping point would the threshold, rather than the system trajectory that results from crossing the threshold). Line 113 uses the term "critical value" but it might be better to note that this is a tipping point or threshold to keep terminology consistent.

We originally gave the definition of bifurcation-induced tipping in line 58 of the text. We agree that it is important to clearly define it since it is important for understanding the novelty of rate-induced tipping. Therefore, now, we first introduce bifurcation-induced tipping in the introduction and we state what a tipping point is:

"This type of tipping is formally associated with the bifurcation of the equilibrium solutions. Bifurcation-induced tipping is caused by reaching a threshold value of a control parameter---here, the intensity of the driver of decline---and that critical value is traditionally seen as the "tipping point"."

Then we also rephrased the introduction of rate-induce tipping. The old "*Rate-induced tipping occurs due to a rate of change in the control parameter, rather than a parameter value as in the case of bifurcation-induced tipping.*" to

"Rate-induced tipping occurs due to a rate of change in the control parameter instead being due to reaching a threshold value of the parameter. Conversely, what matters in bifurcation-induced tipping is the absolute value of the parameter, not how slowly or rapidly that value was reached."

We agree that it is a good idea to keep the terminology more consistent. Therefore, we rewrote line 113 and after to mention more clearly the tipping point:

"At a critical value of the driver of decline, the pollinator community undergoes bifurcation-induced tipping. At this tipping point, the pollinator community collapses---all pollinator species go extinct (very low abundance). By lowering the driver of decline, the system has another bifurcation-induced tipping point, where it recovers at the point of recovery where the first species are reintroduced."

- Line 298: The authors say they run simulations from the "extinct state" but if a species is identically at zero abundance, it seems that it can't recover? Are the authors using the fact that the abundances are starting from a value above numerical zero (which might cause numerical issues) or is the algorithm working differently than my reading suggests?

Thanks for pointing this out. The abundances do not indeed go fully to zero. Equation 1 was missing a factor μ for both plant and pollinator species which we added now to the equations and the text. This μ is a migration rate which is taken to be small. This way, extinct species can recover under the right environment. The parameter μ is set to 0.0001 which is smaller than the extinction threshold of 0.01 we used. See also the sensitivity analysis in the Supporting Information showing that the value of μ has a negligible influence on the dynamics of the model as long as $\mu \ll 0.01$.

We added the migration component to the main text too in a section of its own in the Model, as well as the point on sensitivity (see also answer below).

- I appreciate that the authors carried out a thorough sensitivity analysis in the supplement; it might be worth a sentence somewhere in the main text to point the interested reader to it.

Again, thank you for pointing this out. We now make sure we mention all the supplements in the main text. We added a reference to the sensitivity analysis at the end of the Results section:

"A Sobol sensitivity analysis in SI5 shows the influence of the model parameters on the feasibility of the networks and the point of collapse. The resource congestion q *and adaptation strength* $\boldsymbol{\nu}$ *have the strongest influence in both cases. Furthermore, the migration rate* $\boldsymbol{\mu}$ *influences the results very little, thereby validating our assumption that this parameter does not significantly influence the dynamics of the model."*
