# Supplementary material for: Adaptive foraging of pollinators fosters gradual tipping under resource competition and rapid environmental change
Source: PLoS Comput Biol. 2024 Jan 9;20(1):e1011762. doi: 10.1371/journal.pcbi.1011762 (PMC10802948; doi:10.1371/journal.pcbi.1011762)
Supplement: Attachment — Submitted filename: pollinator_communities_revision.pdf [file pcbi.1011762.s004.pdf]

"Early irreversible collapse of perturbed pollinator communities under rapid environmental change"

The authors have undertaken a very extensive revision of the work and the framing of the results and their implications has markedly improved. In particular, it is now much clearer that the consequences of adaptive foraging constitute the primary novelty, while rate-induced-tipping is a feature of the modeling framework. Clarifications regarding how the model works and results were generated have helped make the results much more understandable overall. I am content with the answers to the more technical queries in my initial review. The abstract, author summary and discussion now also frame the contribution properly and qualify the results more appropriately.

I think the paper could be acceptable for publication in its revised form but I have some additional questions and comments for the authors to consider. As such, I think another minor revision may be warranted.

Comments/questions:

- Figure 1: d_A^{recovery} seems to be mislabeled in panel C here. Also, this panel could be referred back to at Line 196 when the definition of bistability is introduced for a visual aid.
- Lines 242-243 (Figure 2B and Figure 3B):
  - The authors say that changes in relative pollinator persistence are "more gradual with adaptive foraging than without adaptive foraging " but, depending on your interpretation of "gradual', I would argue that the opposite is true. The magnitude of the slope of the curve in the adaptive case is larger across most of the figure so the change is faster, except for around $\theta \approx 0.7$ where the threshold exists in the non-adaptive case. I would consider rewording and/or reinterpreting this part of the text.
  - It would be good to have some interpretation for the increased sensitivity in the adaptive case at lower values of θ, although I think this is partially answered later around Line 303. Also, are the differences/overall trends discussed here sensitive to the values of ν and q?
- Figure 5:
  - The text explaining this figure (Line 268 onwards) needs to do a better job of walking the reader through each part of the figure, rather than asking the reader to digest the figure all at once. To this end, the figure should have panel labels to allow the text to point to parts of the results in more manageable chunks.
  - Some of the context for this figure, and the results contained in it, need to be in the text (this is mostly in the caption currently). For example, we have $\lambda = 0.05$ here but this is useful for the reader to know since it situates us relative to what we saw in Figures 2 and 3.
  - The y-axes in rows 2 and 3 are all different and this leads to some readability issues. My conclusions from looking at these panels definitely changed somewhat once I realised this.

Minor comments/corrections:

- Line 164: The sensitivity analysis is first mentioned here (I think) but there is no instruction of where the reader should find it.
