# Supplementary material for: Adaptive foraging of pollinators fosters gradual tipping under resource competition and rapid environmental change
Source: PLoS Comput Biol. 2024 Jan 9;20(1):e1011762. doi: 10.1371/journal.pcbi.1011762 (PMC10802948; doi:10.1371/journal.pcbi.1011762)
Supplement: Attachment — Submitted filename: Point-by-point reply to reviewers.pdf [file pcbi.1011762.s005.pdf]

Editors' comments:

Thank you very much for submitting your manuscript "Adaptive Foraging of Pollinators Fosters Gradual Tipping under Resource Competition and Rapid Environmental Change" for consideration at PLOS Computational Biology. As with all papers reviewed by the journal, your manuscript was reviewed by members of the editorial board and by two independent reviewers.

Based on the reviews, we are likely to accept this manuscript for publication, providing that you address the additional suggestions/questions of the referees. There is room for improvement in the writing in the transitions between sections and in removing repetitions throughout the text.

We critically reviewed the text, made simplifying language edits, and improved transitions where we could. In the introduction and discussion we strengthened the connection between paragraphs. Furthermore, the following section connectors were added.

End of the Model section, before Methods:
"The model just described sets the foundation for a series of simulations. Next, in the Methods section, we delve into the specifics of how we operationalized these, detailing the parameters and scenarios under which the simulations were conducted."

End of Methods, before Results:
"We used these methods to test the predictions of the model under various conditions. By systematically altering key parameters and observing the results, we aim to unravel the complex dynamics of pollinator communities in response to environmental stressors. The following Results section presents the outcomes of these simulations, shedding light on the practical implications of our model."

End of Results, before Discussion:
"The above results provide a detailed account of how pollinator communities react under different stress scenarios, highlighting the critical role of adaptation and the rate of environmental changes. In the Discussion, we interpret these findings in a broader context, discussing their implications for ecological theory, pollinator conservation strategies, and future research directions."

Reviewer 1

This paper has been extensively revised, and it is now much clearer in terms of focus and objectives. The novelty compared to previous studies is also better highlighted.

We thank the reviewer for their positive assessment and final comments on the text.

It is great to have included the model in the main text. Regarding the structure of the Method's part, I would have one Model & Methods part but this is a personal preference (and I am not sure what is the typical structure for Plos Comp Biol papers).

To minimize the necessary structural changes that occurred throughout the review process, we will keep the separation into these (very related) sections.

One minor thing:
l. 60-61 : « However, most of the theoretical work on pollinator communities has found evidence for the potential for tipping points 23,25–29, especially those based on Lotka-Volterra type of dynamics. »

I would say 'some theoretical work' because you cannot reduce the large number of theoretical studies of plant-pollinator models to these few ones studying tipping points.

We agree with the reviewer and changed "*most*" with "*some*". To better clarify what previous work showed, we further changed this sentence to read: *"However, some of the theoretical work on pollinator communities has shown that tipping points are possible, especially in models based on Lotka-Volterra type of dynamics (Latty2019, Dakos2014, Lever2014, Meng2021, Jiang2019, Huang2021)."*

Reviewer 2

Please see the attached PDF for more detailed comments. The revision has addressed the main issues and improved the paper considerably. I have some additional minor suggestions and questions.

The authors have undertaken a very extensive revision of the work and the framing of the results and their implications has markedly improved. In particular, it is now much clearer that the consequences of adaptive foraging constitute the primary novelty, while rate-induced-tipping is a feature of the modeling framework. Clarifications regarding how the model works and results were generated have helped make the results much more understandable overall. I am content with the answers to the more technical queries in my initial review. The abstract, author summary and discussion now also frame the contribution properly and qualify the results more appropriately.

I think the paper could be acceptable for publication in its revised form but I have some additional questions and comments for the authors to consider. As such, I think another minor revision may be warranted.

We appreciate the positive comments. We have addressed the additional comments and questions below.

Comments/questions:

• Figure 1: drecovery seems to be mislabeled in panel C here. Also, this panel could be referred back to at Line 196 when the definition of bistability is introduced for a visual aid.

We agree that the point of recovery appears to be mislabeled in Figure 1C. The “driver of decline dA” is actually the quantity of interest and does not denote the point of recovery. We agree this is a bit confusing and therefore we redesigned the labels in Figure 1C. Now, we clearly denote the point of recovery.

• Lines 242-243 (Figure 2B and Figure 3B):

- The authors say that changes in relative pollinator persistence are “more gradual with adaptive foraging than without adaptive foraging ” but, depending on your interpretation of “gradual”, I would argue that the opposite is true. The magnitude of the slope of the curve in the adaptive case is larger across most of the figure so the change is faster, except for around $\theta \approx 0.7$ where the threshold exists in the non-adaptive case. I would consider rewording and/or reinterpreting this part of the text.

It is indeed true that, in the adaptive case, the change in persistence takes place over a larger range of theta than in the nonadaptive case. What we meant by “gradual” is that the slope (the derivative) is smaller in the adaptive case. We reworded this in the text to clarify better what we meant, the whole paragraph now reads:

“*Adding adaptive foraging to the model changes the response to the rate of change, but rate-induced tipping is still present (Figure 3B). For low magnitudes of the maximum driver of decline, adaptive communities already experience partial, and sometimes even full, extinction. There is a decrease in relative pollinator persistence over a large region of the fraction θ of the point of collapse d_A collapse (Figure 3B). In comparison, in the nonadaptive case (Figure 2B), there is a more clear threshold around θ ~ 0.7 where pollinator communities go from relatively high persistence to collapse. Adaptive pollinator communities are, thus, more likely to collapse partially over a range of maximum decline drivers rather than exhibit a complete collapse of all species at a specific value of the driver (see Figure S7 for the complete bimodal distributions of pollinator persistence over the networks showing partial collapse). This suggests that adaptive pollinator communities might show a more gradual tipping process in which a few species go extinct at a time rather than a single collapse. Later, we show which species are affected throughout the gradual tipping process.*”

- It would be good to have some interpretation for the increased sensitivity in the adaptive case at lower values of θ, although I think this is partially answered later around Line 303. Also, are the differences/overall trends discussed here sensitive to the values of v and q?

This is indeed mostly answered around line 303 and Figure 7, where we show how the bifurcation points change as a function of the resource congestion q. Similarly, there are figures in the SI showing how changing the value of v influences the tipping points of the pollinator communities. We added a more clear interpretation of the increased sensitivity in the adaptive case, see also the answer to the previous comment. This is also explained more in-depth in Figure 5, where we now add a more clear connection with low theta values:

“*This representative simulation also gives an explanation for the curve in Figure 3B where at low θ values the relative pollinator persistence already decreases before reaching 0 between 0.4 < θ < 0.6. As Figure 5A illustrates, some pollinator species go extinct faster than others (faster relates to a lower driver of decline value). In combination with Figure 3, where the simulations ran until equilibrium, it shows that partial collapse of the pollinator community takes place when there is adaptive foraging.*”

In general, we constrained ourselves by looking at the cases where the pollinator communities possess bistability as given in Figure 7. This constrains the values of v and q we can use. Overall, the trends are qualitatively similar in these ranges of n and q.

• Figure 5:

- The text explaining this figure (Line 268 onwards) needs to do a better job of walking the reader through each part of the figure, rather than asking the reader to digest the figure all at once. To this end, the figure should have panel labels to allow the text to point to parts of the results in more manageable chunks.

We added panel labels to figure 5 to be able to reference more clearly to this figure. We also improved the text and walked through the different parts of the figure. The text surrounding the figure is now split into two paragraphs. We also added an extra paragraph to relate the simulation of figure 5 to the results of figure 3, where there is a partial collapse of pollinator communities at low θ values.

“*To better illustrate how adaptive foraging alters the dynamics under increasing stress, Figure 5 shows a representative simulation of plant and pollinator abundances under increasing drivers of decline d_A. The plant and pollinator species start with a low abundance (S_init = 0.1). The driver of decline d_A is increased from 0 with a rate of lambda = 0.05. Given the slow increase in the driver of decline d_A, both plant and pollinator species initially recover their abundances (Figure 5A and B). Most plant and pollinator species reach their peak around 10 time units after which their abundances decrease. Eventually, all pollinator species go extinct.*

During this process, pollinators shift their foraging effort so that it benefits them the most. Panels C-H of Figure 5 show how their foraging effort changes, sorted by their number of connections (degree). After roughly 10 time units, the driver of decline increases to such a level that many species start to deteriorate. At this point, some large changes occur in the foraging effort. Most notably, a few pollinators manage to invest heavily in one or two plant species, thereby temporarily offsetting their decline in abundance. This leads some plant species to stabilize their decline for an extended period, which allows certain high-degree pollinator species to persist significantly longer than other, lower-degree pollinator species. The two pollinator species with degree 9 (Figure 5H) even temporarily benefit from the extinction of other species, as can be observed by a temporary increase in their abundances (Figure 5A). They increase their foraging effort significantly in one of their connected plant species, leading to a loss of biodiversity in plants while simultaneously promoting two plant species (Figure 5B). At the same time, many low- to mid-degree pollinator species do not manage to adapt their foraging effort in such a way

as to offset the negative effect of the driver of decline. Eventually, the pressure of the driver of decline gets so high that all pollinator species go extinct.

This representative simulation also gives an explanation for the curve in Figure 3B where at low θ values the relative pollinator persistence already decreases before reaching 0 between 0.4 < θ < 0.6. As Figure 5A illustrates, some pollinator species go extinct faster than others (faster relates to a lower driver of decline value). In combination with Figure 3, where the simulations ran until equilibrium, it shows that partial collapse of the pollinator community takes place when there is adaptive foraging."

- Some of the context for this figure, and the results contained in it, need to be in the text (this is mostly in the caption currently). For example, we have $\lambda = 0.05$ here but this is useful for the reader to know since it situates us relative to what we saw in Figures 2 and 3.

We added more of the context and results of the figure to the text. We addressed this in the answer to the previous comment. We added the starting conditions to the text: "*The plant and pollinator species start with a low abundance (S_init = 0.1). The driver of decline d_A is increased from 0 with a rate of lambda = 0.05.*"

- The y-axes in rows 2 and 3 are all different and this leads to some readability issues. My conclusions from looking at these panels definitely changed somewhat once I realised this.

It is indeed difficult to compare the different graphs with different y-axes in rows 2 and 3. To make the comparison between these figures easier, we have given them all the same y-axes. Now, it is clear that the biggest change in adaptive foraging happens in the more generalist pollinators (higher degree).

Minor comments/corrections:

• Line 164: The sensitivity analysis is first mentioned here (I think) but there is no instruction of where the reader should find it.

This is indeed the first time that the sensitivity analysis is mentioned. We now added a reference to the Supporting Information here: "*This assumption is validated through a sensitivity analysis (see SI5).*"
